# The human adenovirus type 5 E1B 55kDa protein interacts with RNA promoting timely DNA replication and viral late mRNA metabolism

**DOI:** 10.1371/journal.pone.0214882

**Published:** 2019-04-03

**Authors:** Berto Tejera, Raúl E. López, Paloma Hidalgo, Reinier Cárdenas, Grisel Ballesteros, Lina Rivillas, Leidys French, Carlos Amero, Nina Pastor, Ángel Santiago, Peter Groitl, Thomas Dobner, Ramón A. Gonzalez

**Affiliations:** 1 Centro de Investigación en Dinámica Celular, Instituto de Investigación en Ciencias Básicas y Aplicadas, Universidad Autónoma del Estado de Morelos, Cuernavaca, México; 2 Instituto de Biotecnología, Universidad Nacional Autónoma de México, Cuernavaca, México; 3 Laboratorio de Bioquímica y Resonancia Magnética Nuclear, Centro de Investigaciones Químicas, Instituto de Investigación en Ciencias Básicas y Aplicadas, Universidad Autónoma del Estado de Morelos, Cuernavaca, México; 4 Institute of Virology, Technische Universität München/Helmholtz Zentrum München, Munich, Germany; 5 Heinrich Pette Institute, Leibniz Institute for Experimental Virology, Hamburg, Germany; University of St Andrews, UNITED KINGDOM

## Abstract

The E1B 55kDa produced by human adenovirus type 5 is a multifunctional protein that participates in the regulation of several steps during the viral replication cycle. Previous studies suggest this protein plays an important role in postranscriptional regulation of viral and cellular gene expression, as it is required for the selective accumulation of maximal levels of viral late mRNA in the cytoplasm of the infected cell; however the molecular mechanisms that are altered or regulated by this protein have not been elucidated. A ribonucleoprotein motif that could implicate the direct interaction of the protein with RNA was initially predicted and tested *in vitro*, but the interaction with RNA could not be detected in infected cells, suggesting the interaction may be weak or transient. Here it was determined that the E1B 55kDa interacts with RNA in the context of the viral infection in non-transformed human cells, and its contribution to the adenovirus replication cycle was evaluated. Using recombinant adenoviruses with amino acid substitutions or a deletion in the ribonucleoprotein motif the interaction of E1B 55kDa with RNA was found to correlate with timely and efficient viral DNA replication and viral late mRNA accumulation and splicing.

## Introduction

The early 1B protein, E1B 55kDa (E1B 55K), from species C human adenoviruses participates in key steps of the virus replication cycle, as it is implicated in the regulation of cellular processes that produce a favorable environment that results in efficient virus gene expression and progeny production [1–3]. E1B 55K has been reported to interact with over 30 proteins, including the viral E4 Orf3, E4 Orf6, DBP, L4 100K, pVI, pVII and a variety of cellular proteins [4]. The role played by the interaction of E1B 55K with each viral or cellular protein in viral replication has not been fully characterized; however, most can be grouped into a few major biological processes that range from regulation of gene expression to polyubiquitin-dependent proteasomal degradation of target proteins. The late phase of infection is characterized by the establishment of a selective expression program in which E1B 55K is required for viral late mRNA intranuclear trafficking, cytoplasmic accumulation [5] and translation [6]. However, E1B 55K is also implicated in the regulation of the anti-viral response of the infected cell. In the absence of this protein, the expression of interferon-sensitive genes like GBP1-5, IFIH1 (MDA5), IFIT2, MX2 and TAP1 increases [7], and its role as inhibitor of the interferon-pathway has been described [8]. A repression domain for interferon target genes has been mapped to the E1B 55K C-terminus [9], while its N-terminus can interact with the tumor supressor p53, tethering a repressor domain that inhibits p53-dependent transcription [10,11]. The interaction with the viral early E4 Orf6 protein and the cellular proteins Cullin 5, Elongins B and C, and Rbx1 is required for assembly of an E3 Ubiquitin ligase complex [12] that promotes polyubiquitylation and degradation of a growing number of cellular substrates that include components of the DNA Damage Response (DDR), such as Mre11, Rad 50 [13,14], the Bloom helicase [15] and DNA ligase IV [16,17], as well as, ATRX [18], p53 [12,19], Tip 60 [20], SPOC 1 [21], Tab182 [22], and α3 integrin [23]. Interestingly, E1B 55K is also responsible for degradation of the PML nuclear bodies component Daxx without the assembly of the E4 Orf6-dependent Cullin5 E3 Ubiquitin ligase complex [24].

Several experiments designed to explore the contribution of specific motifs or domains to the protein’s activities have identified features such as a nuclear export signal (NES) between residues 83 to 93 [25] and a nuclear localization signal (NLS) in the C-terminus [26] that allow shuttling of the protein between the nucleus and the cytoplasm. The protein is modified posttranslationally by phosphorylation at Ser490, Ser491, and Thr495 [27,28], and by sumoylation at Lys104 [29]. Phenotypic analyses of mutant viruses with amino acid substitutions in these sites indicate they impact E1B 55K functions. Phosphorylation is necessary for the proper localization of the protein in the cell nucleus and for its ability to associate with, inhibit and induce proteasomal degradation of p53 [12,28,30], while E1B sumoylation regulates intranuclear targeting and nuclear export of the protein [31], and it has been proposed that it functions as E3 SUMO1 ligase for p53 [32]. E1B 55K is required for efficient accumulation of viral late mRNA and the concomitant inhibition of accumulation of cellular mRNA in the cytoplasm [33]. Since it was reported that the protein associates with the cellular hnRNP E1B-AP5 [34], which interacts with the cellular mRNA export receptor Nxf1 [35], it was suggested that the protein could directly alter cellular mRNA export mechanisms; however, no evidence has been found for the participation of hnRNP E1B-AP5 in the selective export of viral late mRNA, or in the reduction of the cytoplasmic accumulation of viral late mRNAs caused by an E1B 55K insertion mutant [36], whose interaction with hnRNP E1B-AP5 is not disturbed [34]. Export of the E1B 55K and E4 Orf6 proteins from the nucleus of infected cells, which depends on the export receptor, Crm1 (Xpo1), does not affect export of viral late mRNA [37], which are exported through Nxf1 [38].

In the absence of E1B 55K, viral late mRNAs do not accumulate efficiently in a soluble nuclear compartment after their dissociation from the nuclear matrix, and previous to their association with the nuclear membrane [5]. These observations suggest that E1B 55K promotes the intranuclear processing of viral mRNA, while simultaneously interfering with that of cellular mRNA [39].

Very few studies have produced information on the structure of E1B 55K. The protein synthezised from a baculovirus expression system analyzed by gel filtration, velocity sedimentation centrifugation, and glutaraldehyde cross-linking assays was reported to form dimers with a nonglobular, elongated conformation [10]. NMR and circular dichroism experiments demonstrated the protein’s N-terminus is intrinsically disordered [40]. Analysis of the E1B 55K amino acid sequence suggests that a central region, from amino acid 215 to 345 may form a hydrophobic core [41] that contains a predicted ribonucleoprotein (RNP) motif, which was shown to interact with RNA *in vitro* in a non-sequence specific manner. In these experiments E1B 55K was over-expressed in *E*. *coli* as a GST fusion protein and residues between Arg284 and Trp289 were directly implicated in the interaction with the viral RNA [42]. However, no interaction was detected in the context of infection suggesting that either the interaction is weak or the *in vitro* experiments do not reflect the ability of the protein to bind RNA within the infected cell [43].

Studies on E1B 55K functions and structure are relevant to understand the contributions this protein makes to the viral replication cycle and because adenovirus mutants that do not express E1B 55K have been proposed as oncolytic agents to be used in combined anti-cancer therapies, as they display selective replication in tumor *versus* normal cells [44,45]. A number of phase II and III clinical trials using oncolytic adenoviruses have been performed [45,46], but the basis for tumor selectivity remains unclear [6,47–49] and E1B 55K-dependent export of mRNA has been proposed to play a determinant role [49]. To gain insight into the role played by the RNP motif of E1B 55K we have evaluated the protein’s interaction with RNA and explored its contributions to viral replication and virus-host interactions. Using newly constructed adenovirus mutants with substitutions in the putative RNP motif and a combination of methodological strategies that integrate NMR, ITC and molecular modelling, we have determined that E1B 55K interacts with viral RNA in the context of the viral infection, and that amino acid substitutions in different positions of the RNP either increase or decrease this interaction. The activity of the E1B 55K-E4Orf6 Cullin 5 E3 Ubiquitin ligase was not abrogated by substitutions in the RNP motif, nor was the production of viral early or late proteins; however, timely viral progeny production and accumulation of viral DNA was altered, as were the levels and postranscriptional processing of viral late mRNA, providing new insight into the mechanistic basis for the role of the interaction of E1B 55K with viral RNA in regulation of viral gene expression.

## Materials and methods

### Cells and viruses

Monolayers of human foreskin fibroblasts (HFF) and 293 cells were grown in Dulbecco’s modified Eagle’s medium (DMEM) supplemented with 10% fetal calf serum (GIBCO-BRL) and 10% Bovine serum (Biowest), respectively, 100 U of penicillin, and 100 μg of streptomycin per ml under a 5% CO_2_ atmosphere at 37°C. The Ad5 2250, which served as wild-type (Ad5 WT), and all E1B 55kDa (E1B 55K) mutant viruses were constructed using a method described previously [50]. Briefly, the H5*pg*4100 served as the Ad5 parent virus for all constructs. The complete E3 transcription unit from Ad5 was inserted into the H5*pg*4100 backbone generating the Ad5 2250, which served as the Ad5 WT for all experiments, and for construction of the E1B 55K substitution or deletion mutants. The C288A, C288S, W289F and Δ284–289 carry nucleotide substitutions (or a deletion in the case of Δ284–289) that were introduced by site-directed mutagenesis into the Ad5 2250 backbone. All recombinant bacmids were partially sequenced to confirm the substitutions or deletion. The Ad5 mutant H*pm*4149 (E1B^−^), which is null for expression of the E1B 55K protein carries four stop codons and was described previously [31]. All viruses were propagated and titrated by fluorescent foci on 293 cells as described previously [51], and a multiplicity of infection (MOI) of 30 Focus Forming Units (FFU) per cell was used in all experiments. Subconfluent HFF cells (at 90% confluence) were used in all experiments.

### Antibodies

The primary antibodies (Ab) used were the mouse monoclonal (MAb) anti-DBP, B6 [52]; the rabbit polyclonal anti-DBP (a kind gift of T. Dobner); the mouse MAb anti-E1B 55kDa, 2A6 [53]; the mouse MAb anti-p53 (DO-1, Santa Cruz Biotechnology); the mouse polyclonal anti-Mre11 (Novus Biologicals); the mouse MAb anti-β actin (Santa Cruz Biotechnology); and the mouse MAb anti-fiber (Abcam). The secondary antibodies used were anti-mouse and anti-rabbit horseradish peroxidase (HRP)-conjugated antibodies (both from Jackson ImmunoResearch), anti-mouse Alexa Fluor 568, and anti-rabbit Alexa Fluor 488 (both from Invitrogen).

### Primers design

Primers were designed using the CLC sequence viewer software and primer-BLAST (NCBI). The primers used to detect the L5 pre-mRNA (L5NP) recognize a region within the L5 primary transcript upstream of the coding sequence, and an amplification product was obtained from nucleotides (nts) 31,007 to 31,123. Primers used to detect the spliced L5 mRNA (L5P) spanned the splice junction between the third exon of the tripartite leader (TPL) and the L5 exon. The forward primer was complementary to the spliced junction (9,720–9,733; 31,042–31,049) and the reverse primer was complementary to the L5 exon sequence (nts 31,189–31,212) (S1A Fig). To measure viral DNA, primers were designed to amplify the second intron in the TPL sequence (nts 7,273–7,353). The latter were also used to perform RT qPCR in the experiments for RNA immunoprecipitation and were previously described [54]. β actin mRNA was used as loading control in the experiments that detected L5NP and L5P. To perform *in vitro* transcription assays primers were complementary to the intron-exon junction between the second intron and the third exon of the TPL (nts 9,601–9,796). The forward primer has a T7 promoter sequence upstream of the complementary sequence. The PCR product served as the DNA template to obtain the corresponding RNA (RNA TPL 196nts). A shorter RNA probe (20nts), whose sequence is included in the RNA TPL 196nts and corresponds to the junction between the second intron and the third exon of the TPL was obtained. The forward primer (used to obtain the RNA TPL 196nts) also served to obtain the 20 nts RNA probe (RNA TPL 20nts) and corresponds to nts 9,632–9,651. Primer sequences are shown in S1B Fig.

### RNA inmunoprecipitation assays

HFF cells were infected with Ad5 WT or the E1B 55K mutants and harvested at 36 hpi. Cell pellets were resuspended in lysis buffer (HEPES 50 mM pH 7.5, NaCl 140 mM, EDTA 1mM, 1% Triton X-100, 0.1% Sodium deoxycholate). Samples were sonicated using a SONIC-Ruptor 4000 (OMNI International), at amplitude of 40% for 60 seconds to lyse the cells. This process was repeated twice. Cell debris was removed by centrifugation at 10,000 g at 4°C for 5 min. All samples were treated with DNAse 10U/μl (Promega) for 15 minutes at 37°C. 10% of the total cell lysate from each sample was used to determine the RNA input. For immunoprecipitation, samples were incubated overnight with the anti-E1B MAb 2A6. Subsequently, protein A-Sepharose was added and incubated for 1h at 4°C. Samples were centrifuged and beads were washed using four consecutive buffers. Buffer 1: 50 mM HEPES pH 7.5, 140 mM NaCl, 1 mM EDTA, 1% Triton X-100, 0.1% Sodium deoxycholate; buffer 2: 50 mM HEPES pH 7.5, 500 mM NaCl, 1 mM EDTA, 1% Triton X-100, 0.1% Sodium deoxycholate; buffer 3: 10 mM Tris HCl pH 8, 250 mM LiCl, 0.5% NP40, 1 mM EDTA; buffer 4: 10 mM Tris HCl pH 8, 1 mM EDTA, 100 mM NaCl. Beads were resuspended in elution buffer (100 mM Tris HCl pH 8, 10 mM EDTA, 1% SDS) and the RNA was isolated using Trizol Reagent according to the manufacturer´s instructions (Invitrogen). Equal volumes of total RNA were analyzed by RT qPCR (Applied Biosystems) using primers complementary to the second intron of the TPL sequence from nucleotides 7,273 to 7,353 (RNA TPL 81nts). RT minus (RT-) controls were included to rule out DNA contamination in the pull-down assays. For the RT- reactions an average C_T_ of 32 was obtained in triplicates from three independent experiments, while the RT+ reactions produced average C_T_ values of 17.

The input for RNA TPL 81nts was quantified and normalized to β actin mRNA levels. These data were then normalized to WT values, obtaining the fold-change for the input RNA between WT and the E1B mutants. The IP data were normalized to the values obtained with the samples from the E1B null virus taken as a measure of the non-specific background. The final values to determine the difference in protein-RNA interaction between WT and E1B mutants were calculated as the percentage of the IP over the input values.

### *In vitro* transcription assays

HFF cells were infected with Ad5 WT and harvested at 36 hpi. Cell pellets were incubated in Tween 20 (1:200)/proteinase K (1mg/ml) at 55°C for 1 hour. The proteinase K was inactivated by incubation at 95°C for 10 minutes. Total DNA was precipitated using 1:10 vol/vol 3M sodium acetate pH 5.2 and 1 vol isopropanol. DNA pellets were resuspended in Tris HCl 10mM pH 7.5 and 100 ng of total DNA were amplified by endpoint PCR (Thermo Scientific) using the forward primer that carries the T7 promoter sequence fused to the sequence that is complementary to the viral DNA described above. The DNA amplicon whose sequence corresponds to an intron-exon junction in the Major Late region between nts 9,601 and 9,796 served as template for T7 RNA polymerase *in vitro* transcription (Ribomax Large Scale RNA Production Kit, Promega). RNA was precipitated using 1:10 vol/vol 3M sodium acetate pH 5.2 and 1 vol isopropanol. RNA pellets were resuspended in nuclease free water and stored at -70°C. This transcript, RNA TPL 196nts, was used in the Nuclear Magnetic Resonance Spectroscopy and Isothermal Titration Calorimetry experiments.

### Peptide synthesis and purification

The peptides that correspond to the region that spans the RNP1 between residues 281 to 289 of the Ad5 WT E1B 55K protein sequence (RGCAFYCCW) and a mutant peptide with substitutions, C287S/C288S (RGCAFYSSW), were synthesized by solid-phase and Fmoc strategy, as previously described [55,56]. All peptides were amidated at the carboxy-terminus. Crude peptides were purified to over 95% purity by reverse-phase high-performance liquid chromatography and characterized by MALDI-TOF.

### Nuclear Magnetic Resonance Spectroscopy (NMR)

NMR spectra of both the WT and mutant peptides were recorded at 25°C on a Bruker 500MHz DRX spectrometer equipped with a 5-mm triple resonance cryoprobe with z-gradients. Two-dimensional [^1^H-^1^H] total correlation spectroscopy (2D TOCSY) (80 ms spin-lock times) were collected on a 0.5 mM peptide solution in 25 mM KCl, 2.5 mM MgCl_2_, and 10% D_2_O, in the absence and presence of 0.156 μM RNA TPL 196nts (nts 9,601–9,796) or RNA TPL 20nts (nts 9,632–9,651)). Spectra were processed and analyzed with NMRPipe and CARA. Most of the residues could be assigned based on the residue type, while the signal corresponding to Cys 283 was identified as the only Cys residue in the mutant peptide. However, it was not possible to distinguish between Cys 287/288 or Ser 287/288 using the available data. We did not observe a signal for Gly 282 HN, but the HA were assigned.

### Isothermal Titration Calorimetry (ITC)

ITC experiments were performed at 25°C on a Malvern ITC200 instrument. Each experiment consisted of 20 injections, each one with 2.0 μl, with injection spacing of 180s. A total of 1.6 M peptide (WT or mutant) was injected into the cell containing 0.156 mM RNA (RNA TPL 196nts or RNA TPL 20nts). All the samples were exchanged into identical buffer to ensure minimal buffer mismatch. To account for the heat of dilution, the background titration, consisting of the identical titrant solution into only the buffer solution, was subtracted. The data were subsequently analyzed with the integrated public-domain software packages NITPIC, SEDPHAT and GUSSI [57–59].

### Western blot assays

To analyze the steady-state concentrations of the cellular proteins, Mre11, p53, β actin and the viral proteins DBP and fiber, HFF cells infected with Ad5 WT or the E1B 55K mutants were harvested at 16, 24 and 36 hpi in 25 mM Tris-HCl, pH 8.0, 50 mM NaCl, 0.5% (w/v) sodium deoxycholate, 0.5% (v/v) Nonidet P-40 (NP-40), and 1 mM phenylmethylsulfonyl fluoride and incubated 30 min at 4°C. Cell debris was removed by centrifugation at 10,000 g at 4°C for 5 min. The cell extracts were analyzed by sodium dodecyl sulfate-polyacrylamide gel electrophoresis (SDS-PAGE) and immunoblotting. β actin was used as the loading control. For immunoblotting, equal amounts of total protein were separated by SDS-PAGE, transferred to PVDF membranes (Millipore), and processed as described previously [41]. Bands were visualized by enhanced chemiluminescence as recommended by the manufacturer (Pierce, Thermo Fisher Scientific) on X-ray films (Kodak). Autoradiograms were scanned and cropped using Adobe Photoshop CC.

## Immunofluorescence

HFF cells grown on glass coverslips to approximately 90% confluence were mock- infected or infected with Ad5 WT or the E1B 55K mutant viruses. The infected cells were processed at the indicated times postinfection, as described previously [36]. After the application of specific primary antibodies, cells were incubated with secondary antibodies coupled to fluorophores as indicated. The coverslips were mounted on glass slides in PBS–10% glycerol, and samples were examined using a Zeiss Axiovert 200M inverted microscope with a 63x/1.4-numerical-aperture oil-immersion objective lens with an Axiocam MRM and Axiovision 3.1 software (Carl Zeiss, Inc.).

### DNA purification and quantitative PCR

Total DNA was isolated from mock-, Ad5 WT- and E1B 55K mutants-infected HFF cells at 16, 24 and 36 hpi. Cell pellets were incubated in Tween 20 (1:200)/proteinase K (1mg/ml) and incubated at 55°C for 1 hour. The proteinase K was inactivated by incubation at 95°C for 10 minutes. Cell debris was removed by centrifugation at 10,000 g at 4°C for 5 min. Total DNA was precipitated using 1:10 (vol/vol) 3M sodium acetate pH 5.2 and 1 vol isopropanol. DNA pellets were resuspended in Tris HCl 10mM pH 7.5 and stored at -20°C. Viral DNA was quantified from equal volumes of total DNA using the Power SYBR Green PCR Master Mix kit according to the manufacturer’s instructions (Applied Biosystems). All primers were validated to confirm an amplification efficiency >90% calculated by the linear regression obtained from standard-curve assays. The primers amplified a unique product of the expected size, as determined by melt-curve analyses. The StepOne system (Applied Biosystems) was used for thermocycling. The DNA samples were analyzed by the standard-curve method using a fragment of the Major Late DNA sequence from nucleotide position 7,007 to 7,480 to calculate the linear regression from the standard curve. The number of DNA copies were plotted as the log of the mean value with standard deviations from triplicate values of two independent expeirments using Prism 7.0 software.

### Splicing assays

HFF cells were infected with Ad5 WT or the E1B 55K mutant viruses and harvested at 36 hpi. Total RNA was isolated using Trizol Reagent according to the manufacturer´s instructions (Invitrogen). Equal volumes of total RNA were analyzed by RT qPCR using the primers for L5P and L5NP. The samples were analyzed by the ΔΔC_T_ comparative method using triplicate samples from two independent experiments. β actin mRNA was used as endogenous control.

### Quantification of IFIT2 mRNA

HFF cells were treated with 1U/ml of human interferon β1 (IFNB1) (Novus biologicals) or with 0.1% BSA for 12 h in DMEM. After this time, cells were mock-infected or infected with the Ad5 WT or Δ284–289 viruses and harvested at 8 and 36 hpi with Trizol reagent (Invitrogen). Total RNA was isolated and equal volumes were analyzed by RT qPCR using primers specific for IFIT2 (ISG54). The samples were analyzed by the ΔΔC_T_ comparative method using triplicate samples from two independent experiments. β actin mRNA was used as endogenous control. IFIT2 was detected with the following primers (5’ to 3’) Fwd: TGTTCCATTCTTGCCAGCC, Rev: CATACCGCAGATGGAGCAG.

### Molecular modeling

The amino acid sequence of the human adenovirus type 5 E1B 55K was aligned with the LH3 protein from a snake adenovirus [60] using the Basic local alignment search tool-NCBI-NIH (Blast), resulting in a significant alignment (24% identity) from residue 164 to residue 349. This region was modeled with I-TASSER [61] using PDB structure 5G5O (LH3 from snake adenovirus) as a template, for both the wild-type and deletion (Δ284–289) versions of the protein; these were achieved with C-scores of 0.40 and 0.57, respectively (scores greater than -1.5 indicate useful models, as detailed in [61] and in the server web page). The resulting models were submitted to the PDB2PQR/APBS servers [62,63] to calculate their electrostatic potentials at pH 7 with CHARMM36 [64] charges and atomic radii. Both models were also submitted to HDOCK [65] to perform docking with a 19 bp segment of dsRNA (PDB structure 1QC0), using default parameters. The 100 predicted complexes with the best interaction energies were further analyzed with VMD [66].

### Protein disorder analysis

For disorder analysis the E1B 55K amino acid sequence of human adenovirus type 5 was used [UniProt ID: P03243] with the following servers: PONDR pool [PONDR VLXT, PONDR XL1_XT, PONDR VL3-BA, PONDR VSL2] (www.pondr.com); IUPRED (https://iupred2a.elte.hu) and DISOPRED (http://bioinf.cs.ucl.ac.uk/psipred_new/).

### Coevolution analysis

The GREMLIN software (http://openseq.org/submit.php) was used to search for coevolving residues with potential structural significance. The HHblits algorithm was used to generate a multiple sequence alignment (MSA) of all available E1B 55K sequences. Sequences having >50% gaps were filtered out.

### Statistical analysis

All data were analyzed in Prism 7.0 software. ANOVA and t-test were used to determine the statistical significance.

## Results

### E1B 55K interacts with RNA in Ad5-infected cells

**Fig 1:**
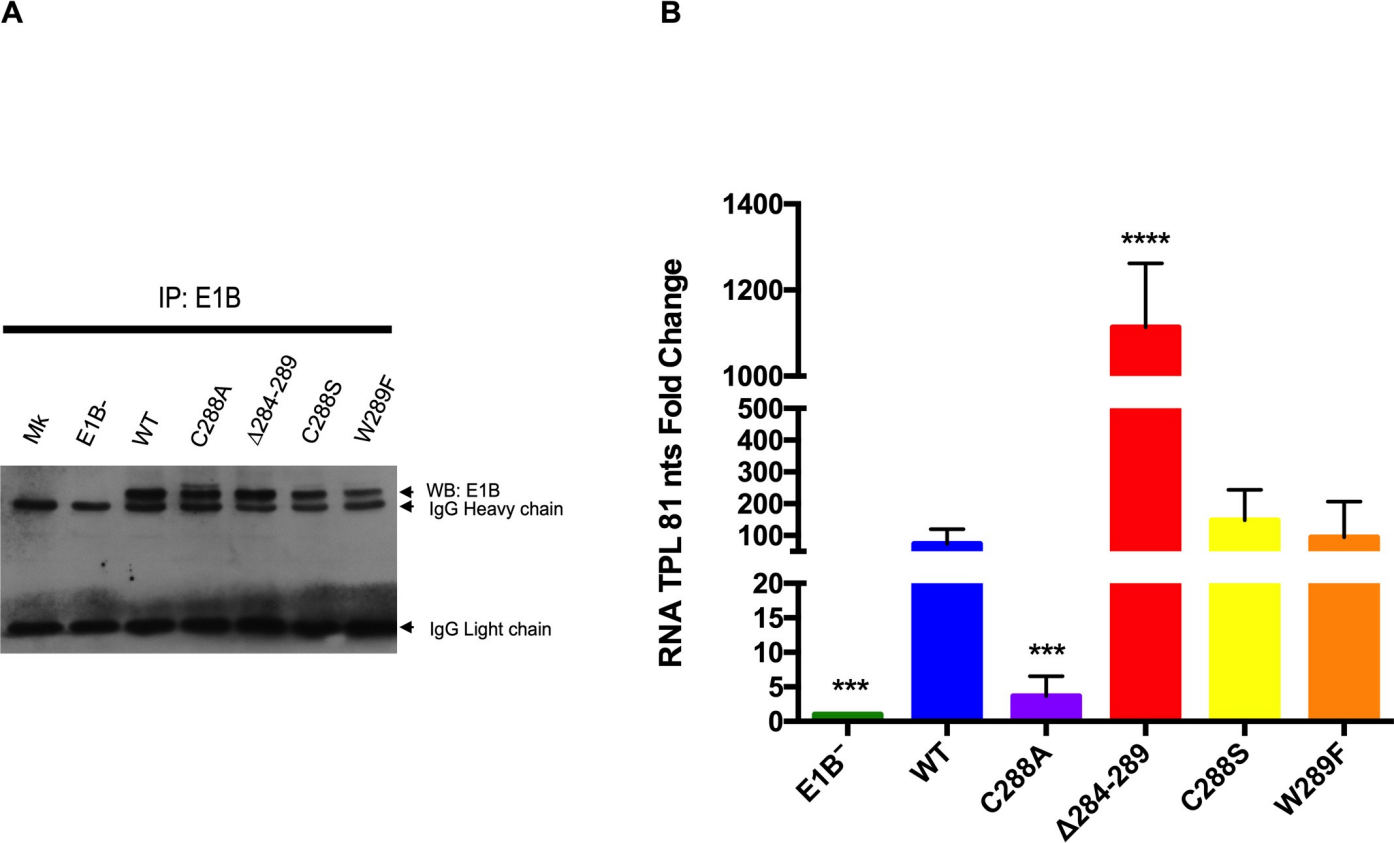
E1B 55K interacts with RNA in Ad5 WT-infected cells and RNP substitutions affect RNA binding. HFF cells infected with the indicated viruses were harvested at 36hpi. E1B 55K was immunoprecipitated with the 2A6 MAb, RNA was isolated and RT qPCR were performed to detect a sequence corresponding to intron 2 in the TPL. (A) Western blot of immunoprecipitated samples with the anti-E1B 55K 2A6 MAb. (B) RT qPCR of immunoprecipitated viral RNA. Immunoprecipitation data was normalized as described in Materials and Methods and it is represented as the percentage of the input RNA. Standard deviations from three independent experiments performed in triplicate are shown. *** P<0.001, **** P<0.0001.

It has been suggested that the interaction of E1B 55K with viral RNA through the RNP motif may be weak or transient during infection. Therefore, to detect a possible interaction between E1B 55K and viral late mRNA in the context of infection, an RNA immunoprecipitation assay (RIP) was performed, in which total protein lysates of cells infected with the Ad5 WT or E1B^−^ viruses or with the RNP mutants C288A, C288S, Δ284–289, and W289F were immunoprecipitated with the anti-E1B 55K MAb-2A6, and the pulled-down RNA were amplified by RT qPCR (Fig 1).

Immunoprecipitation of the E1B 55K protein was observed in similar amounts in lysates from cells infected with the Ad5 WT virus, as well as with all the mutants tested. Moreover, no protein expression was observed in lysates of cells infected with the E1B^−^ virus that does not direct the synthesis of E1B 55K (Fig 1A). Once the protein was immunoprecipitated, the viral late RNA associated was amplified by RT qPCR (Fig 1B). The differences in the amount of RNA recovered from cells infected with the Ad5 WT and E1B^−^ was nearly 100-fold, demonstrating that under the conditions tested E1B 55K associates with the viral late RNA (Fig 1B). The RNA sequence amplified corresponds to the TPL RNA of 81nts (S1 Fig), which was selected because all five families of viral late mRNA from the adenovirus major late transcription unit contain this sequence in their 5'-noncoding region and it is necessary for their efficient export from the nucleus and translation [67, 68, 69]. Even though similar levels of the E1B 55K protein were immunoprecipitated from the lysates of cells infected with Ad5 WT virus or each of the E1B 55K-RNP1 mutant viruses (Fig 1B), the amount of RNA amplified from each sample was significantly different. The RNA pulled-down from the C288A mutant was nearly 20-fold lower than in Ad5 WT, an expected result considering the reduced binding of this E1B 55K protein to RNA previously reported [42]. Interestingly viruses that harbor the subtitutions C288S and W289F displayed only minor variations in their ability to associate with RNA. In sharp contrast, over 10-fold higher amount of RNA was recovered with the Δ284–289 mutant compared to Ad5 WT. The latter result was unexpected, as deletion of the RNP eliminates the residues that were previously suggested to participate in the direct contact of the RNP with RNA [42]. Nevertheless, taken together these results indicate that E1B 55K can interact with RNA in infected cells, and the interaction is affected by substitutions in the RNP sequence.

### An E1B 55K RNP1 peptide binds RNA *in vitro*

**Fig 2:**
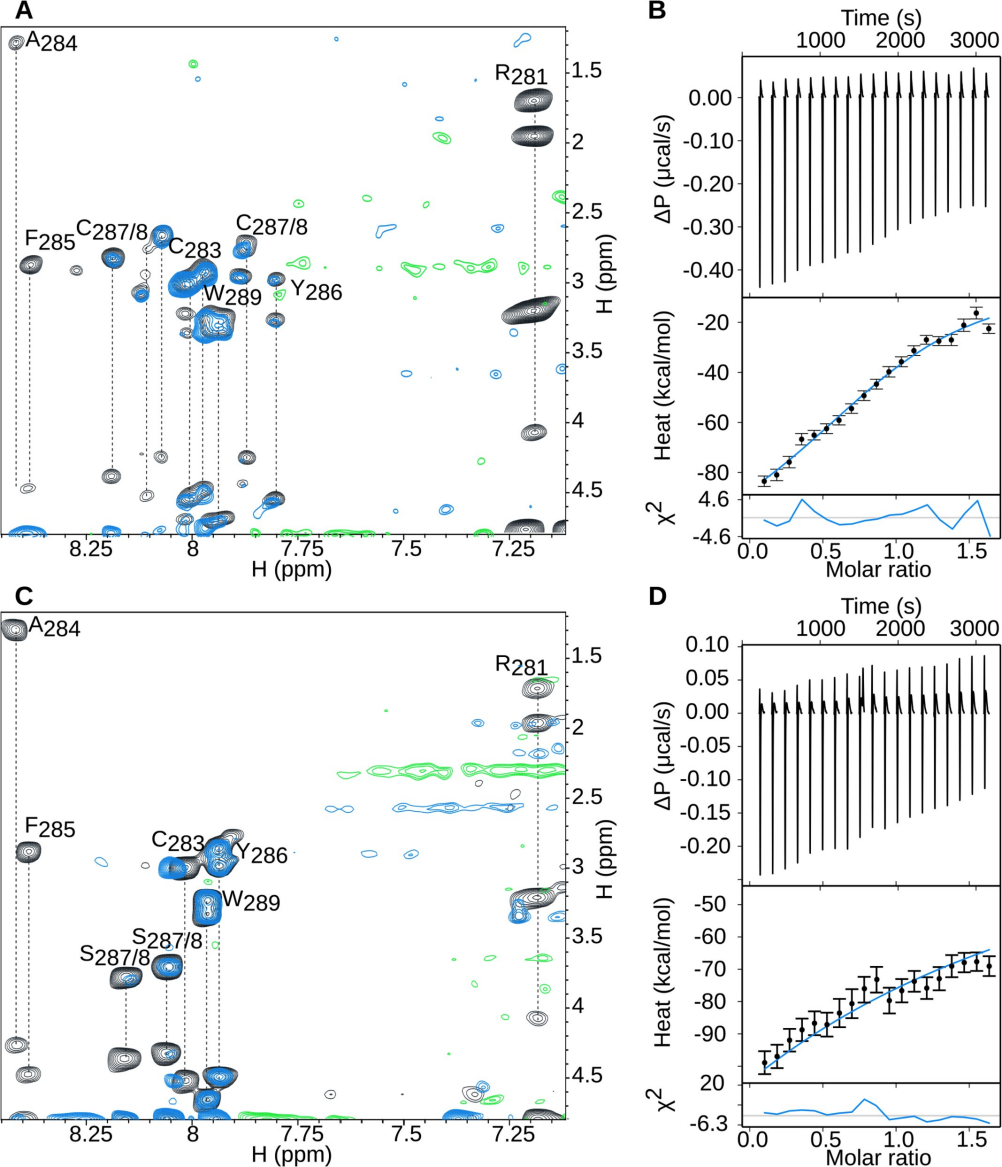
E1B 55K-RNP peptides interact with TPL RNA 196nts *in vitro*. **(**A) Expanded region of an overlay of TOCSY spectra of free WT peptide (black) and WT peptide bound to the TPL RNA 196nts probe (blue). **(**B) Heat exchanged from each injection of WT peptide into a solution containing the TPL RNA 196nts probe. (C) Expanded region of an overlay of TOCSY spectra of free C287S/C288S peptide (black) and C287S/C288S peptide bound to the TPL RNA 196nts (blue). (D) Heat exchanged from each injection of C287S/C288S peptide into a solution containing the TPL RNA 196nts. The thermograms were best fit to a one binding site model.

Previous studies of the E1B 55K-RNA interaction determined *in vitro* showed that substitutions in the E1B 55K-RNP sequence either increased or decreased binding of the protein to RNA [42]. When residues A284S and F285Ldel287 (in which C287 was deleted) were substituted the resulting E1B 55K proteins showed reduced RNA binding, while the C288A substitution displayed only minor reduction, and W289F showed increased binding [42], suggesting that these residues may either directly participate in RNA contact or contribute to the proper conformation of the RNP motif. To determine whether the RNP motif may participate in the direct interaction with RNA, we evaluated if a synthetic peptide corresponding to residues 281 to 289 of the E1B 55K polypeptide can bind RNA *in vitro*, using NMR and ITC. The TPL RNA 196nts probe that contains the TPL intron 2 –exon 3 junction of the tripartite leader (S1 Fig) was synthesized as described in Materials and Methods and used for these experiments. The sequence was selected because all five families of viral late mRNAs from the adenovirus major late transcription unit contain this sequence in their 5'-noncoding region [68,69]. Two peptides were tested in the NMR and ITC experiments: a peptide with the Ad5 WT (WT) sequence (RGCAFYCCW) and a peptide with substitutions in C287S and C288S (RGCAFYSSW), termed C287S/C288S peptide, in which the cysteine residues were substituted to avoid disulfide linkage formation, and because their substitution (C288A) or deletion (F285Ldel287) affect RNA binding [42]. The WT and C287S/C288S peptides were titrated with the TPL RNA 196nts probe and the interaction was followed by NMR and ITC, as described in Materials and Methods (Fig 2).

The chemical shift difference between the TOCSY spectra of both peptides, with and without RNA, indicates residues whose chemical environment has been affected by the binding. The shifts obtained indicate that both peptides interact with the RNA, and significantly that the most affected residues were R281, A284 and F285 in both peptides (Fig 2A and 2C). Furthermore, the ITC titration confirmed RNA binding for both peptides (Fig 2B and 2D), with slightly higher heat exchanges for the WT peptide, which displayed an exothermic process that suggests a higher affinity interaction. The binding of WT and mutant peptides was also tested against a 20 nts RNA (TPL RNA 20nts) probe whose sequence is included in the TPL RNA 196nts and corresponds to the intron-exon junction; however, in this case the NMR spectra and the ITC results indicate that there was no binding (S2 Fig). These results are in agreement with Horridge and Leppard [42], who showed that substitution of C288A has a mild effect and that A284S or F285LdelC287 severely reduce binding of E1B 55K to RNA. Our data agree with these observations and further suggest that A284 and F285 may participate in direct contact of the protein with RNA.

### Substitutions in the E1B 55K-RNP alter timely progeny production

**Fig 3:**
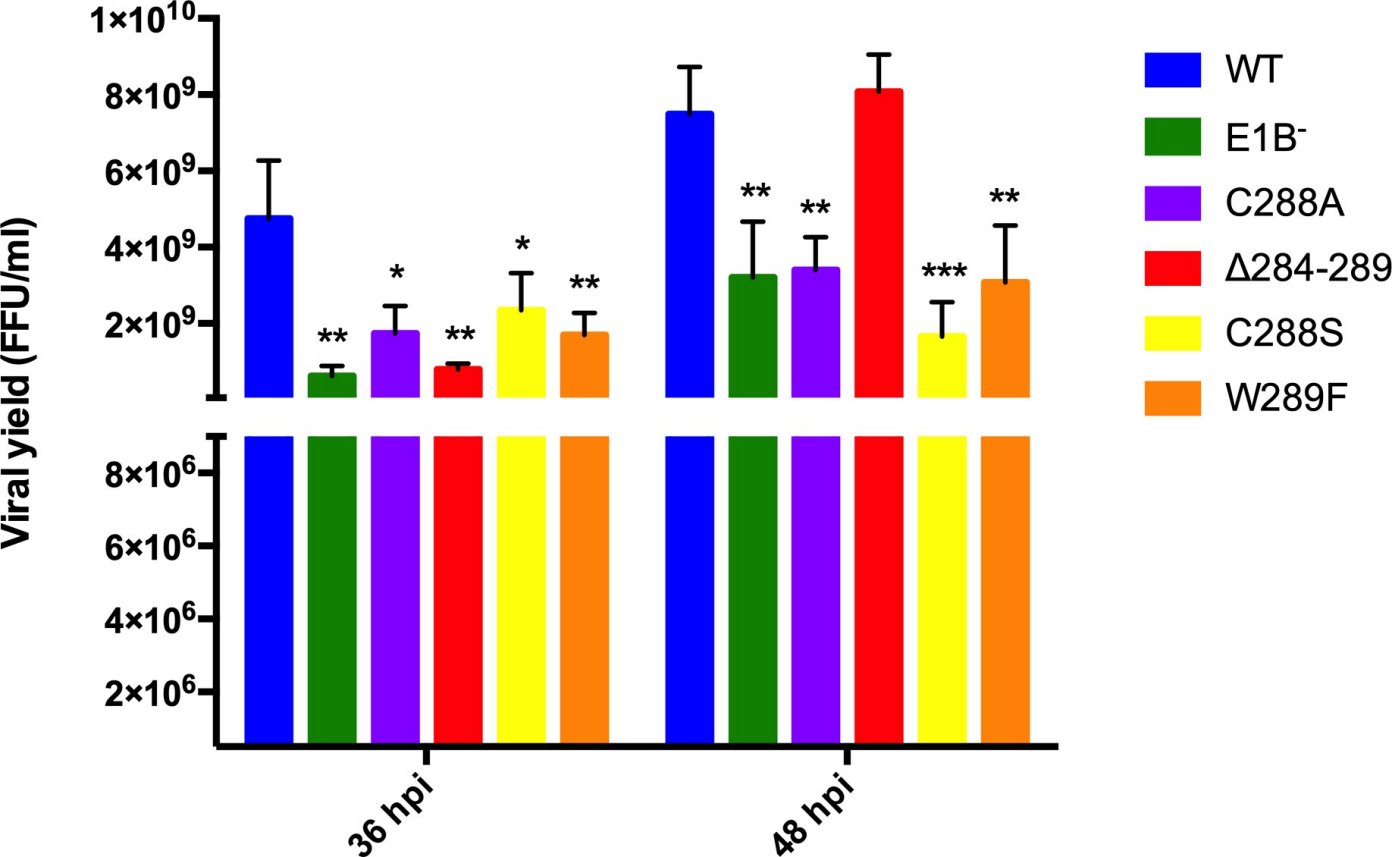
Effect of substitutions in the E1B 55K RNP motif on viral progeny production. HFF cells were infected at a MOI 30 FFU/cell and harvested at 36 and 48 hpi. Viral titers were determined in 293 cells by fluorescent foci using a mouse monoclonal anti-E2 72K (DBP) antibody. The standard deviations from two independent titration experiments are shown. * P <0.05, **P<P0.01, ***P<0.001.

E1B 55K is a multifunctional protein implicated in various key processes during viral replication that ultimately affect viral progeny production. To determine the effect of the E1B 55K-RNP substitutions on virus replication, mutant viruses were analyzed for their ability to produce viral progeny compared to the wild-type virus. Infected cells were harvested at late times post-infection (36 and 48 hpi) and viral titers were determined by immunofluorescence (Fig 3).

All mutant viruses showed defects in viral progeny production at 36hpi compared with Ad5 WT. Interestingly in the case of the Δ284–289 mutant virus the defect was slightly more severe at this time-point compared with the other E1B 55K RNP mutants. In fact, this reduction in viral yield was similar to the defect observed in the E1B^−^ mutant. The decreases in viral yield were statistically significant, and were similar to those reported for other RNP mutants [43]. However, while for the C288 and W289 mutants this phenotype was maintained (C288A, W289F) or was slighltly more severe (C288S) at 48 hpi, the Δ284–289 mutant produced viral progeny at levels that were comparable with Ad5 WT, indicating that mutations in the RNP motif alter the efficieny and the timely production of progeny.

### Mutations in E1B 55K-RNP1 do not impair the E1B 55K/E4 Orf6—E3 Ubiquitin ligase activity

**Fig 4:**
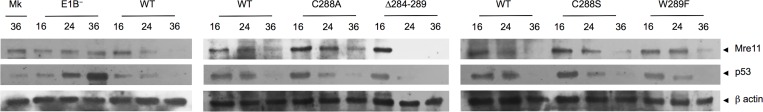
E1B 55k RNP substitutions do not impair degradation of Mre11 and p53. HFF cells were infected and harvested at 16, 24 and 36 hpi. Total protein extracts were obtained and Western blot assays were performed employing anti-Mre11 (Novus Biologicals) and anti-p53 DO1 (Santa Cruz Biotechnology) antibodies. β actin (Santa Cruz Biotechnology) was used as the loading control.

Since mutations in the E1B 55K coding sequence that prevent the assembly of the E3 Ubiquitin ligase complex result in a decrease of viral yield [36], we decided to determine if the altered progeny production observed with the RNP mutants (Fig 4) correlate with impaired activity of the E1B 55K/E4Orf6—E3 Ubiquitin ligase. HFF cells were infected and harvested at times post-infection that were previously established [36] to correspond to early (16 hpi), transition to late phase (24 hpi) and a late time post-infection (36 hpi), and the levels of Mre11 and p53 proteins were compared between Ad5 WT and the RNP motif mutants (Fig 4).

As expected, a clear decrease in the levels of Mre11 and p53 could be observed in Ad5 WT-infected cells by 36 hpi, and when E1B was not expressed (E1B^−^), failure to assemble the E3 Ub-ligase resulted in increased p53 levels and no change was observed in Mre11 levels. Cells infected with C288A, C288S or W289F mutant viruses, displayed similar levels of Mre11 and p53 at the different times post-infection compared with the levels observed in Ad5 WT-infected cells. Interestingly, in the case of the Δ284–289 mutant, p53 and Mre11 levels decreased to undetectable levels by 24 hpi, suggesting that their degradation initiated earlier or proceeded more efficiently than in Ad5 WT-infected cells. These results indicate that although deletion of the RNP motif resulted in earlier decrease of the selected cellular protein targets, the activity of the E3 Ubiquitin ligase was not impaired by the E1B RNP mutations in agreement with previous observations [43].

### Effect of E1B 55K-RNP substitutions on viral early or late protein levels

**Fig 5:**
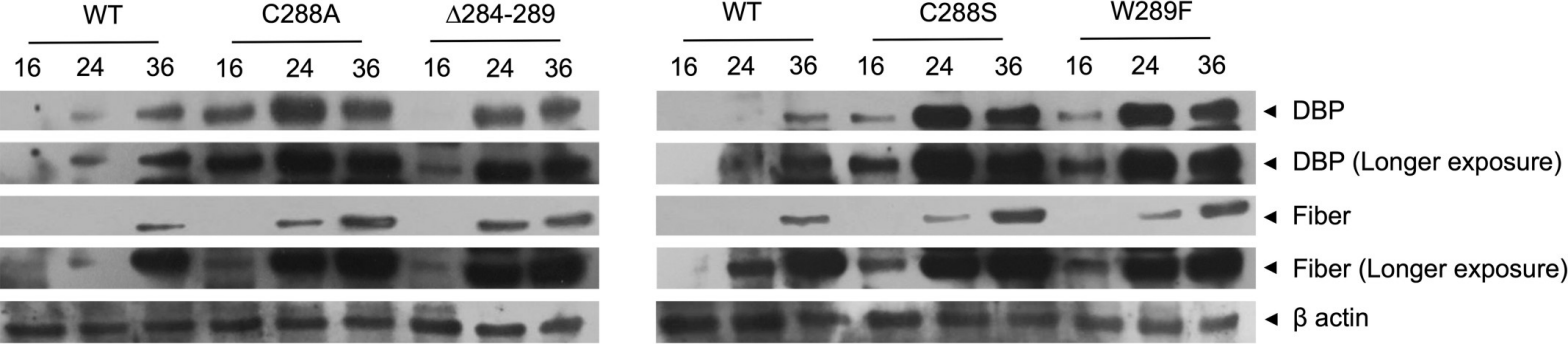
Effect of substitutions in the E1B 55K RNP on kinetics of accumulation of viral proteins, DBP and fiber. HFF cells infected with Ad5 WT or E1B 55K mutants were harvested at 16, 24 and 36 hpi. Total protein extracts were obtained and western blot assays were performed using the anti-DBP (B6) and anti-fiber (Abcam) antibodies. β actin (Santa Cruz Biotechnology) was used as the loading control.

One of the primary defects of adenovirus mutants that do not express the E1B 55K is the reduced synthesis of viral late proteins, which results from decreased accumulation of viral late mRNA in the cytoplasm; therefore, we decided to evaluate if the E1B RNP mutations impact the accumulation of fiber protein. In these experiments the early DBP protein was also included. The steady state levels of both proteins were compared between Ad5 WT and the RNP motif mutants in total cell lysates harvested at 16, 24 and 36 hpi by Western blot assays (Fig 5).

In Ad5 WT-infected HFF cells the DBP and fiber proteins were detected at 24 and increased by 36 hpi. In the case of fiber, the protein could only be detected at the earlier time-point in longer exposures of these blots. In contrast, shorter exposures were used for comparison with the RNP mutants, as higher protein levels were detected. Thus, in all cases, the levels of both DBP and fiber increased as the viral replication cycle progressed, but higher levels of DBP were obtained in all mutants compared with those observed from Ad5 WT. In the case of fiber, the protein was detected earlier in the mutants than in Ad5 WT infected cells, although the levels were only moderately higher. These unexpected results suggest that substitutions in the E1B 55K RNP motif do not abrogate viral early or late gene expression but affect their timely expression.

### Mutations in the RNP motif do not impair E1B 55K intranuclear localization

**Fig 6:**
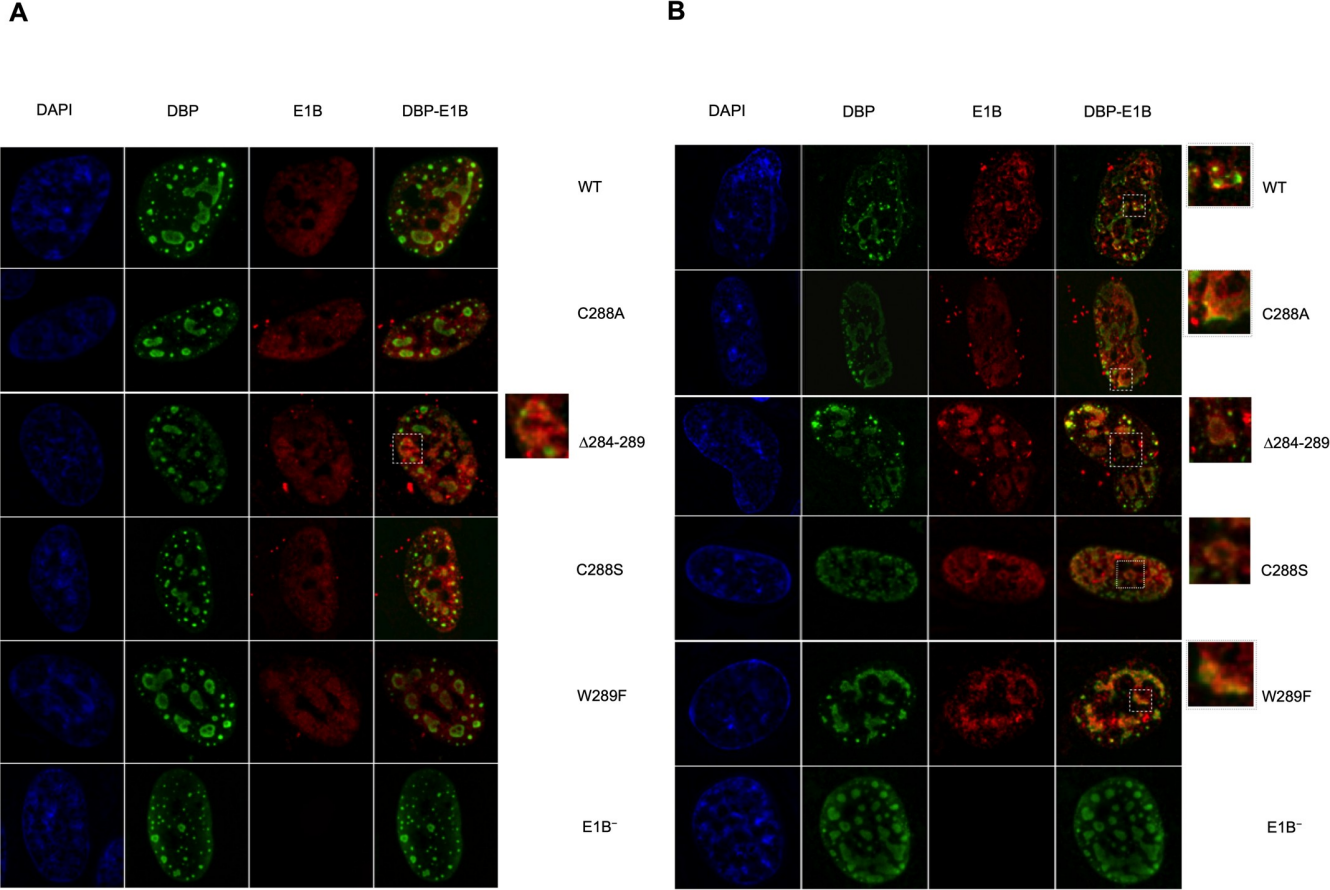
Localization of DBP and E1B 55K to viral RC in Ad5 WT- or RNP mutants-infected HFF cells. HFF cells infected with Ad5 WT or E1B 55K mutant viruses were fixed and processed for immunofluorescence as described in materials and methods. Blue (DAPI), green (DBP), red (E1B 55K). (A) 24hpi, (B) 36hpi. Results shown are representative of at least two independent experiments.

The timely localization of E1B 55K in viral Replication Compartments (RC)—intranuclear sites where the viral genome is replicated and expressed—is known to be required for efficient viral DNA replication [8], viral late mRNA biogenesis and ultimately viral progeny production [5,36,39,70]. To determine if the E1B 55K RNP motif mutations affect its subcelular localization, particularly in the RC, HFF cells infected with the Ad5 WT and mutant viruses were analyzed at 24 and 36 hpi by immunofluorescence microscopy (Fig 6).

In these experiments the viral DBP protein was used as a *bona fide* component of viral RC. In Ad5 WT-infected cells the DBP is known to be distributed forming structures with a doughnut-shaped or ringed appearance, and as the transition to the late phase of viral replication progresses (after 24 hpi), E1B 55K colocalizes more extensively with DBP (Fig 6A). The ring-shaped structures then seem to coalesce at later times of viral replication. The Ad5 WT and E1B mutant viruses showed the expected DBP pattern, with a ring-like distribution that coalesced by 36hpi (Fig 6B). E1B 55K-DBP colocalization was more evident at 36hpi and no discernible differences were observed comparing Ad5 WT and E1B mutant viruses. These data suggest that the E1B 55K RNP motif mutations do not impair the intranuclear distribution of E1B 55K.

### Timely viral DNA replication is altered by subtitutions in the E1B 55K RNP motif

**Fig 7:**
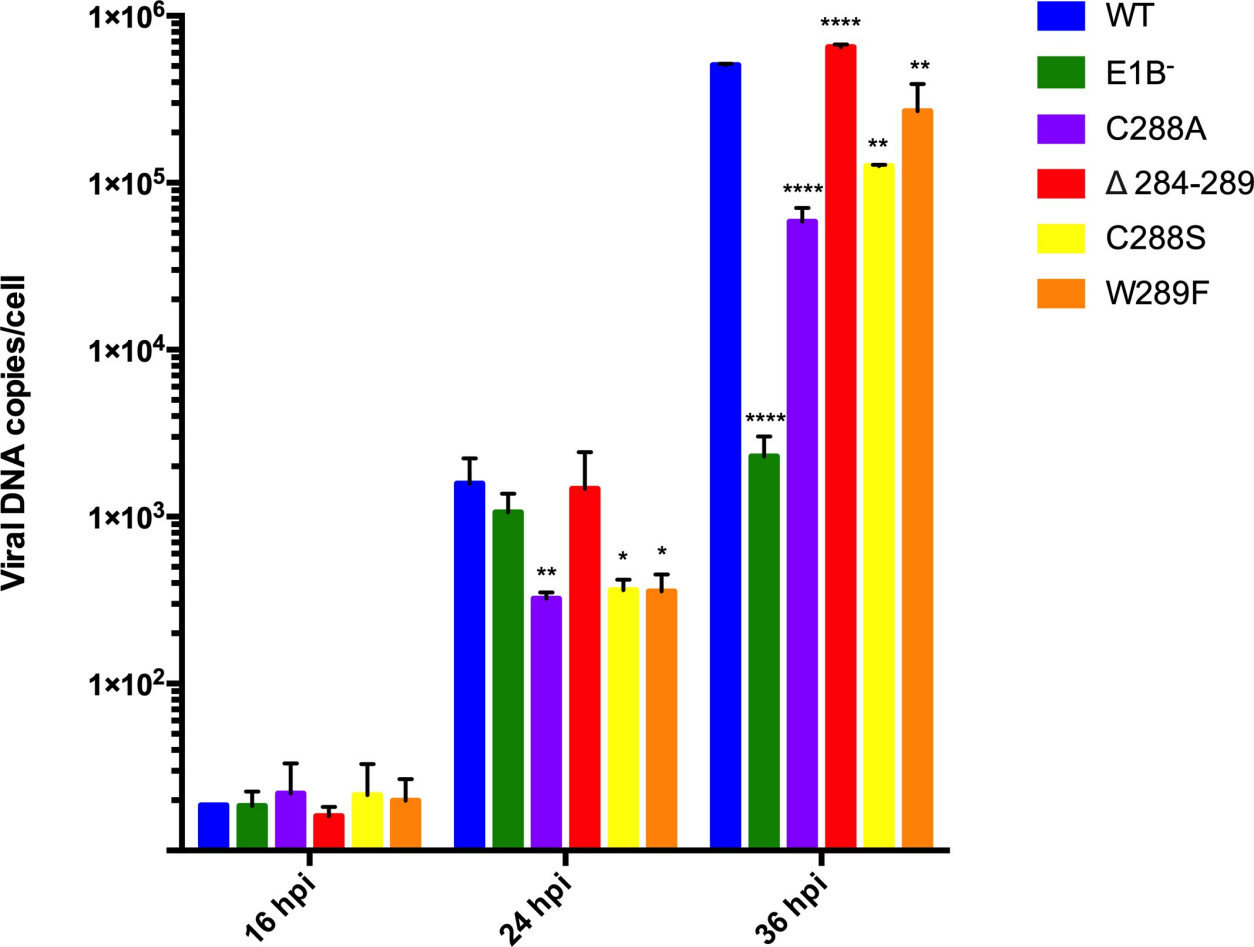
Kinetics of viral DNA accumulation are altered by mutations of the E1B 55K RNP motif. HFF cells infected with Ad5 WT or E1B 55K mutants were harvested at 16, 24 and 36 hpi and total DNA was isolated. Viral DNA was amplified through a quantitative PCR and a viral DNA absolute quantification was performed. Data are shown as viral DNA copy number per cell of duplicate samples from two independent experiments. * p<0.05, ** p<0.01, ** p<0.001, **** p<0.0001, t-test.

Since mutations in the E1B 55K RNP reduced viral progeny production (Fig 3), but did not abrogate the activity of the E3 Ub ligase (Fig 4), changes in the timing and level of viral protein expression (Fig 5) could be the result of altered timing or efficiency of viral DNA replication. Therefore, qPCR were performed to determine the kinetics of viral DNA accumulation measured at various time-points of the viral replication cycle (Fig 7).

The expected pattern of DNA accumulation was observed in Ad5 WT-infected HFF cells [36]. The input level (16 hpi) was initially detected, followed by 10- and 1000-fold increases in the viral DNA copy number at 24 and 36 hpi, respectively (Fig 7). Also as expected, the E1B 55K null virus displayed delayed and reduced (about 100-fold lower) levels of viral DNA by 36 hpi. Interestingly, the RNP mutants showed varying degrees of altered timing and levels of viral DNA. Statistically significant differences between these viruses and Ad5 WT were observed by 24 hpi, and lower levels of viral DNA than the Ad5 WT virus were produced by 36 hpi in cells infected with the C288A, C288S and W289F mutants. These results correlate with the reduced efficiency of progeny production in these viruses. In contrast, Δ284-289-infected cells produced similar levels of viral DNA compared with Ad5 WT by 24 and 36 hpi.

### Increased E1B 55K-RNA binding correlates with higher steady-state levels and splicing of the L5 mRNA

**Fig 8:**
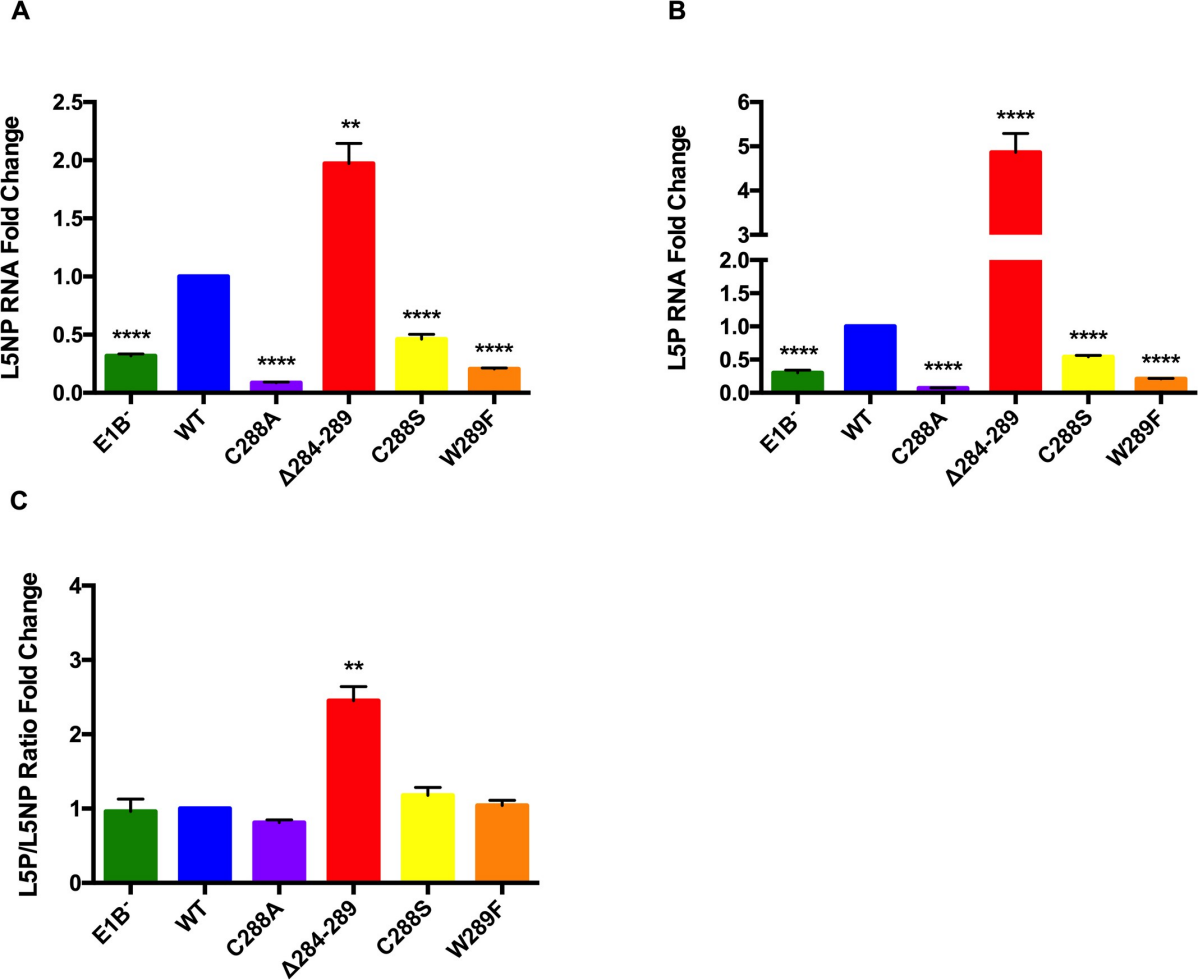
Substitutions in E1B 55K RNP affect viral late mRNA biogenesis. HFF cells infected with Ad5 WT or E1B 55K mutants were harvested at 36 hpi and total RNA was isolated. Viral late pre-mRNA levels were determined for L5 RNA by RT qPCR against an (A) intron-exon (L5NP) or (B) Exon-exon (L5P) junction, for the unspliced and spliced L5 mRNA species, respectively. (C) To compare the splicing efficiency the L5P:L5NP ratios were calculated. β actin mRNA was used as endogenous control. Data from two independent experiments performed in triplicate are shown. ** P<0.01, ****P<0.0001.

The altered kinetics of viral DNA replication between Ad5 WT and the RNP motif mutants suggest that the interaction of E1B 55K with RNA may also impact the expression of viral genes at different levels. To determine a possible role of the E1B RNP motif on viral late mRNA biogenesis, the steady-state levels of both viral late pre-mRNA and mature mRNA were quantified by RT qPCR, and their ratio was calculated as a measure of splicing efficiency. Total RNA was obtained from Ad5 WT- and RNP mutants-infected HFF cells and primers that differentiate the unspliced (L5NP) vs the spliced (L5P) form of the L5 mRNA transcript were quantified, as described in Materials and Methods (Fig 8).

The absence of E1B 55K (E1B^−^) resulted in 3-fold reductions of both the unspliced and spliced L5 mRNA, and no discernable effect could be observed on splicing efficiency, as the ratio of L5P/L5NP mRNA were similar to Ad5 WT levels. Similar, 2-fold reductions in the levels of both the L5NP and L5P mRNA were observed for the C288S mutant virus, while the mutants C288A and W289F displayed 14 and 5-fold reductions, respectively (Fig 8A and 8B). As with the E1B^−^ mutant, none of these substitutions affected splicing efficiency (Fig 8C). An unexpected finding was that L5 mRNA levels were 2 to 5-fold higher in cells infected with the RNP motif deletion mutant Δ284–289, an effect that was opposite to what was expected, given the negative effect observed for the C288 and W289 substitutions. Moreover, in contrast to the E1B^−^ and the C288 or W289 mutants, deletion of the RNP resulted in 2.6-fold higher levels of spliced over unspliced L5 mRNA, indicating that both higher mRNA steady state levels and more efficient splicing were induced when residues 284 to 289 were deleted from E1B 55K.

### Bioinformatic analysis and molecular modeling of the interaction of E1B 55K with RNA

**Fig 9:**
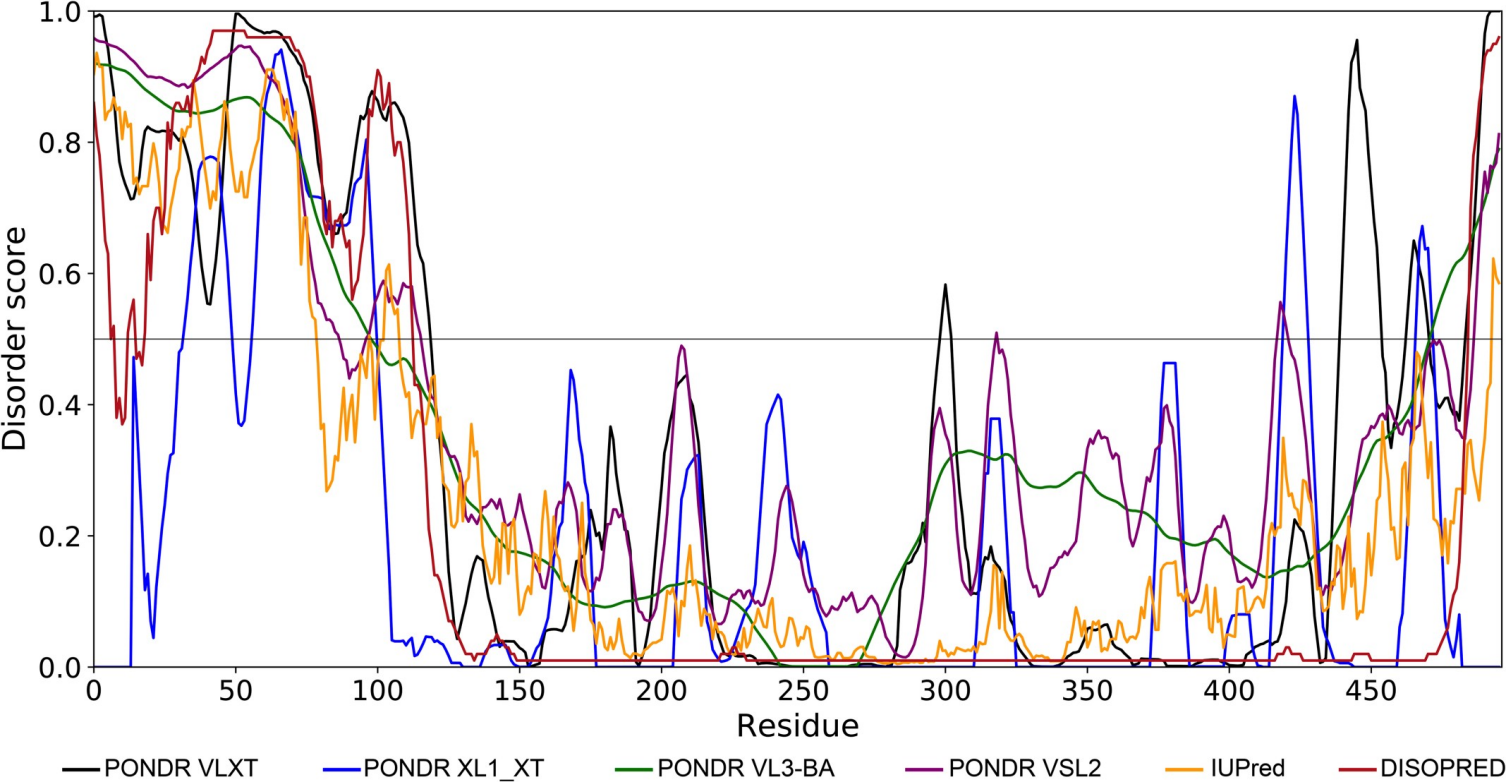
Intrinsic disorder in E1B 55K. PONDR VLXT, PONDR XL1_XT, PONDR VL3-BA, PONDR VSL2, IUPred and DISOPRED predictors were used for disorder analysis. All predictors indicate a high level of intrinsic disorder in the N- and C-terminus.

As described in the introduction, the putative RNP motif lies within a conserved region that may represent a hydrophobic core flanked by less conserved N- and C-termini, where most of the functional regions of the protein have been mapped. The N-terminus has been shown to be intrinsically disordered [40], but no information is available on the structure of the central or C-terminal regions. Using several predictors of intrinsic disorder it was confirmed that residues from position 1 to 150 at the N-terminus display high scores of disorder. Also, most of the algorithms predicted that residues from 390 to 496 at the C-terminus display relatively high scores for disorder, while residues from approximately 150 to 380 display a relatively high degree of order, suggesting that the protein may be organized in three discernible structural domains (Fig 9).

**Fig 10:**
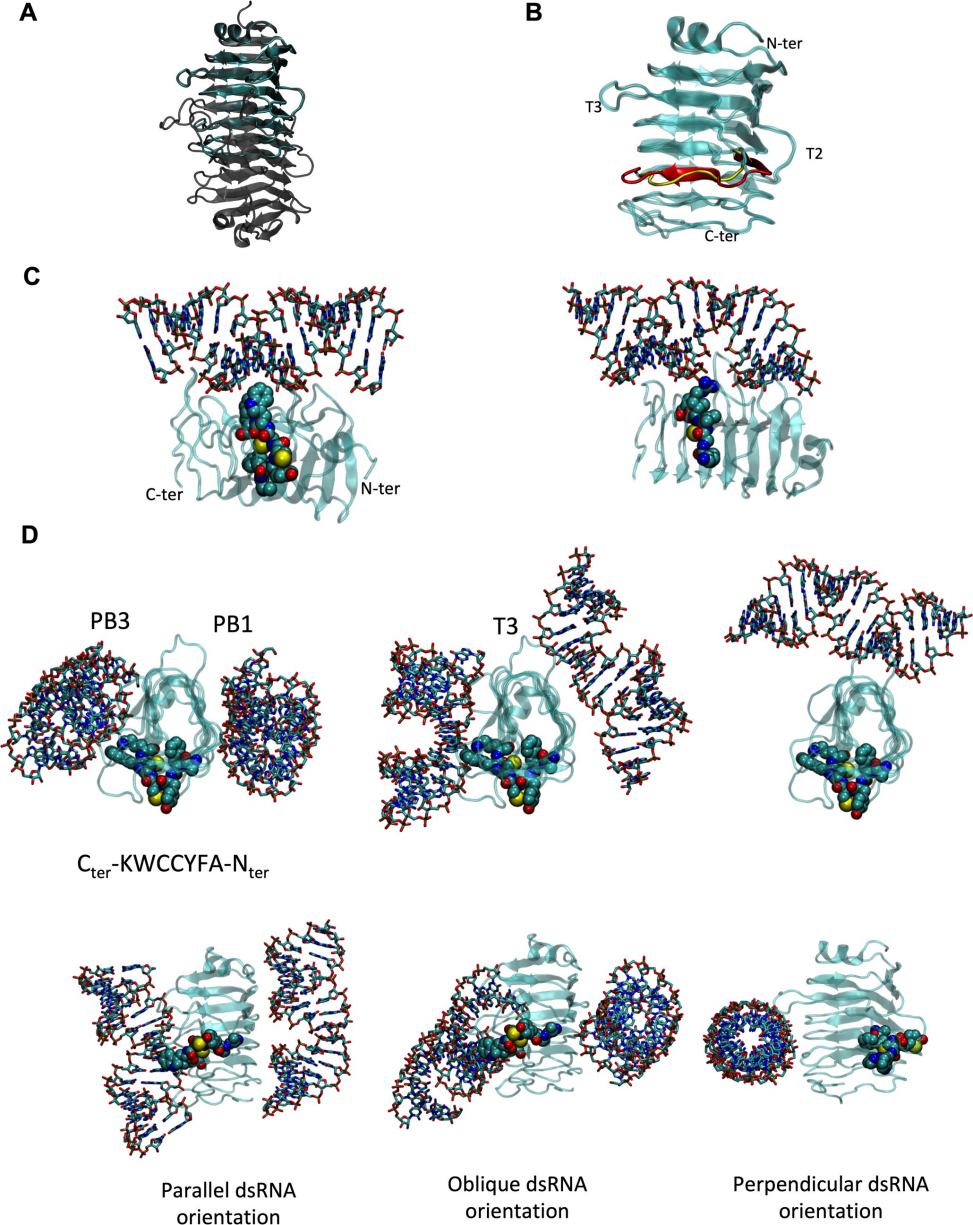
E1B 55K-RNA interaction model. (A) Superposition of the model for the wild-type central domain that contains the putative dsRNA binding motif (solid cyan ribbons) and the parent LH3 structure (transparent gray ribbons). (B) Superposition of the models for the wild-type (cyan and red ribbons) and deletion Δ284–289 (cyan and yellow ribbons) versions of the putative dsRNA binding motif of E1B 55K, in the same orientation as above facing the surface designated as PB3. N- and C-termini, as well as loops belonging to T2 and T3 surfaces are indicated. PB2 lies at the right of the figure and PB1 at the back, following the nomenclature of LH3. (C) Interaction of dsRNA with the putative RNA-binding motif in E1B 55K. Left: best ranked complex for the wild-type protein. Right: best ranked complex for the mutant protein. dsRNA is shown in sticks, the protein domain in a translucent ribbon (N-terminus to the right) and the RNP motif in spacefilling representation, carbon in cyan, nitrogen in blue, oxygen in red, sulfur in yellow. (D). Sample of the classes of RNA-protein conformations or poses found for the wild-type domain, showing different angles of interaction between dsRNA and the long axis of the domain. The first column shows two views, rotated 90 degrees, of the interaction with PB3 and PB1 surfaces with a parallel register of the domain and RNA main axes. The second column shows two views, rotated 90 degrees, of the interaction with PB3 and PB1 surfaces and the T3 loop with an oblique register of the domain and main axes. The third column shows the interaction with the T3 loop in a perpendicular register of the domain and the main dsRNA axis. The protein is depicted as a translucent cyan ribbon, with the RNA binding domain in a spacefilling representation and CPK colors. dsRNA is shown in sticks with CPK colors (C in cyan, N in blue, O in red, S or P in yellow).

In order to rationalize the effects of the mutants in the context of the full E1B 55K, we modeled its ordered region using LH3 from a snake adenovirus [60]. While sequence identity is low (24%), we consider this a valid template because of its equivalent position in the adenovirus genome of Atadenovirus and Mastadenovirus, which suggests a common ancestor. Furthermore, the quality of the models for the wild-type and Δ284–289 proteins is adequate for their use as working models (a superposition with the parent structure is shown in Fig 10A), and the structure is stabilized by short ladders and clusters of hydrophobic residues (S3 Fig), typical of beta-helices.

**Fig 11:**
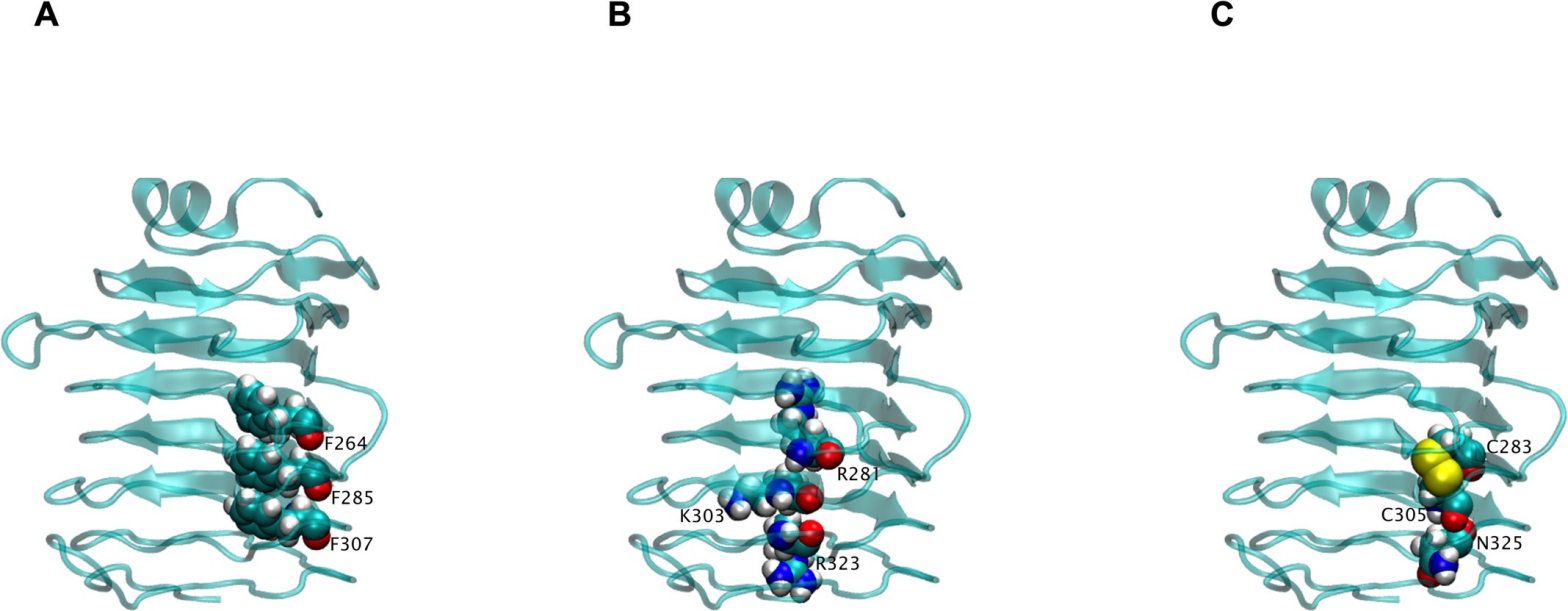
Representation of amino acids with highest probability of coevolution on the E1B 55K model. Three-dimensional model of the E1B 55K central region with VDW representation of amino acid with highest probability of coevolution. A) F264-F285-F307. B) R281-K303-R323. C) C283-C305-N325.

Analysis of coevolution between amino acid residue positions of all reported E1B 55K sequences using the Gremlin server further supported the possibility that the protein core is organized as a β solenoid structure because residues between amino acids 148 and 383 showed higher scores, especially in paired residues that interact in the core structure, while very low scores were obtained for residues outside of this region (shown as bold in the S1 Table). Analysis of the positions on this model of the residues that were predicted to coevolve (Fig 11) showed that phenylalanines 264, 285 and 307, which may be involved in the stabilization of the solenoid structure (S3 Fig) display a similar ladder to those on the LH3 protein [60] (Fig 11A). Positively charged residues, R281, K303, R323, occupy the same side of the beta solenoid, creating a highly positive region (Fig 11B), while C283, C305 and N325 also seem to interact (Fig 11C), lending support to the idea that the central domain folds into a beta solenoid strutcure.

The superposition of both models is shown in Fig 10B, highlighting that the RNP deletion has a local effect on the structure. This could explain why there are no drastic phenotypes in protein degradation and/or protein localization assays. A surface representation in S4 Fig shows that the RNP lies at the T2 loop and PB3 face; the deletion results in placing an additional positive charge roughly at the same position as K290, adjacent to the C-terminus of the putative RNP. The electrostatic potentials mapped on the molecular surface are shown in S5 Fig, for the three main surfaces of the domain (PB1, PB2, and PB3, as defined by Menendez et al [60]). Again it can be observed that the effect of the deletion is local, and that the electrostatic potential is similar for both versions.

To model the protein-RNA interaction, the TPL RNA 81nts detected in pull-down assays or the TPL RNA 196nts probe used in NMR and ITC were approximated as a dsRNA of 19bps. We chose this particular conformation as a secondary structure prediction of TPL showed that these structures were possible. The best binding poses are shown in Fig 10D, displaying superficial and non-specific interactions of both dsRNA grooves. Most importantly, W289 and K290 emerge as important residues in the interaction, providing a rationale for the modest decrease in interaction for the W289F mutation. C288 is located in the core of the domain, in keeping with the modest effect of the C288S mutation; the C288A mutation could be destabilized compared to the wild-type protein, as C288 forms part of the cystein ladder in the core of the protein (S3 Fig). dsRNA can bind at various surfaces of the protein, as shown in Fig 10C and S6–S8 Figs. This allows for simultaneous binding of two dsRNA regions, which could be important for promoting mRNA processing and/or organization of protein-RNA complexes in the infected cell. Upon analysis of the 100 best-energy poses for each protein, we found that those of the wild-type version can be classified in a few groups, as shown in Fig 10C. On the other hand, the deletion mutant displayed a much larger collection of poses, prototypes of which can be seen in S6–S8 Figs. This could be related to the intriguing finding of an increase in RNA binding of the RNP deletion mutant compared to the wild-type version, where an increase in affinity would be explained with an entropic argument, instead of a better direct interaction.

## Discussion

The E1B 55K protein makes several contributions to the viral replication cycle; however, the molecular mechanisms that are altered or regulated by this protein in the infected cell are incompletely understood. Here we have shown that E1B 55K interacts with viral RNA in the infected cell (Fig 1) and that the RNP motif can participate in direct protein-RNA contacts (Fig 2). Substitution of amino acid residues at positions in the RNP motif that were previously shown to either reduce or increase RNA interaction *in vitro* [42] displayed similar patterns of altered binding, confirming that the RNP and the positions that were substituted are relevant for the interaction in infected cells. Nevertheless, deletion of the RNP motif increased RNA binding, indicating that its exclusion results in changes that promote the interaction. Interestingly, molecular modeling of E1B 55K with dsRNA suggests the protein can associate with two such molecules and that the deletion of the RNP may increase the conformational arrangements that the E1B 55K-dsRNA complexes can adopt (Fig 10).

The multifunctional nature of E1B 55K is likely to depend on postranslational modifications and on the variety of molecular interactions the protein engages. Such molecular interactions can be expected to determine both the protein’s intracellular localization and activities, which are likely to be interdependent. Phosphorylation of the E1B 55K C-terminus promotes the protein’s SUMOylation, a modification that increases its localization in the viral RC [31,71], a site where the protein is likely to exert its role on viral DNA replication and viral gene expression [70], as well as on viral mRNA processing. Other activities, such as the assembly of the E1B 55K/E4 Orf6-dependent E3 Ubiquitin ligase may depend on nucleoplasmic localization or translocation of the protein to the cytoplasm. The interactome of E1B 55K in infected cells has been reported [4], and although it is not clear how each of the reported molecules may influence the protein localization or activity, at least some of the interactions can be expected to occur at different times of the viral replication cycle and different subcellular sites. Consequently, different subpopulations of the E1B 55K may exist during the viral replication cycle, as the protein engages different interactions and activities. The results in this work indicate that one such interaction is with viral late mRNA, and that changes in E1B 55K-RNA binding alter phenotypes that are implicated in the normal progression of the viral replication cycle.

Substitutions in the RNP altered the efficiency or timely production of viral progeny. In the case of the C288A, C288S and W289F mutants, levels of viral progeny were comparable to those produced by the E1B null virus, both at 36 and 48 hpi. In contrast, the Δ284–289 mutant displayed reduced virus production only at 36 hpi, as by 48 hpi virus production reached levels that were similar to Ad5 WT (Fig 3). Lower efficieny or delayed virus production could originate from changes in viral gene expression, viral DNA replication, degradation of protein targets, adequate formation of viral RC, or virus assembly, and all but the latter are known to require E1B 55K. Since degradation of p53 and Mre11 was not abrogated by susbtitutions in the RNP motif E1B 55K-RNA binding is not implicated in assembly of the E1B 55K-E4 Orf6-dependent E3 Ubiquitin ligase (Fig 4). Rather, viral gene expression and DNA replication seem to be directly affected (Figs 7 and 8). Interestingly, the C288A, C288S and W289F mutants displayed severely reduced viral DNA replication at 24 hpi—at levels that were 3-fold lower than the E1B null virus, suggesting a dominant negative effect. In contrast, the Δ284–289 mutant displayed no such defect at this time-point. However, while from 24 to 36 hpi the increase in viral DNA copies in the E1B null virus was minimal, all RNP mutants displayed increments that were comparable to Ad5 WT (Fig 7), suggesting that all but the deletion RNP mutant display a defect in the initial replication of viral DNA, and that the E1B 55K produced by the RNP mutants can support WT levels of viral DNA replication at later time-points of infection. Such an effect could be related to the timely role of E1B 55K on formation of viral RC, which in turn impacts efficient viral DNA replication [8], and to the recently described bi-phasic kinetics of viral genome replication [72]. Although immunofluorescence experiments showed no clear effect on E1B 55K or DBP localization in RC (Fig 6), it is possible that mutations that affect the RNP motif may hinder E1B 55K activities even when the protein can associate with RC, where RNP mutations may affect the early low rate, but not the late high rate of DNA replication.

Nevertheless, differences in the efficiency and timing of DNA replication are not sufficient to explain the low levels of progeny produced by the C288S, C288A and W289F mutants, suggesting an additional defect in these viruses that was not displayed by the deletion mutant (Fig 3). Although no evidence has been reported for participation of E1B 55K in viral DNA encapsidation or assembly, viral proteins implicated in viral packaging are associated with the periphery of viral RC [73]. Moreover, the intriguing finding that the E4 Orf6 protein is associated to the virus surface and may function as a portal for genome packaging [74] further suggests E1B 55K may affect viral packaging. Therefore, it will be of interest to determine whether E1B 55K-RNA binding may alter the proper organization of RC, as well as the interaction with E4 Orf6 which may affect virus assembly.

Since E1B 55K is implicated in the regulation of the anti-viral response, the RNP motif mutant’s phenotypes may stem from failure to timely inhibit cellular defense mechanisms, such as the expression of interferon-stimulated genes (ISG) [7, 8]. This was indeed the case for the RNP deletion mutant, which displayed decreased repression of IFIT2, a previously reported E1B 55K-repressed ISG [7, 8] (S9 Fig), and it will be of interest to determine whether E1B 55K-RNA binding may be required for transcriptional repression of p53- or IFN-dependent genes.

Other antiviral mechanisms could be affected and should be evaluated, such as the regulation of the death-associated Daxx factor [24] or regulation of KAP1, each of which may also impact viral progeny production [75]. However, since degradation of p53 and Mre11 was not abrogated by deletion of the RNP motif but repression of IFIT2 was defective, these findings further indicate that the activities of the E1B 55K/E4 Orf6-dependent E3 Ubiquitin ligase are unrelated to E1B 55K activities in ISG repression.

All RNP mutants showed anticipated timing and enhanced accumulation of the early DBP and late Fiber proteins (Fig 5), but it is not clear whether this may alter the program of viral or cellular gene expression and result in delayed progeny production. To our knowledge, a direct effect of E1B 55K on viral early gene expression has not been demonstrated, and it will be interesting to determine whether the protein and the RNP motif may be implicated in regulation of early genes. In contrast, maximal levels of viral late mRNA require E1B 55K [3, 5]. Interestingly, increased E1B 55K-RNA binding in the deletion mutant correlated with higher than Ad5 WT levels of the L5 viral late mRNA production and splicing (Fig 8). Although we have evaluated the effect of mutations in the RNP motif on different phenotypes during productive infection, in our experiments the interaction of E1B 55K with RNA was evaluated only at 36 hpi and it will be of interest to determine the effect of the interaction at other times of viral replication on viral early and late gene expression. Nevertheless, the 10-fold increase in RNA binding observed for the RNP deletion mutant correlated with more efficient production of viral late mRNA, lending further support to the notion that the protein participates in viral late mRNA production [5, 39], and providing a rationale for the mechanistic basis in which the interaction of E1B 55K with RNA is necessary for intranuclear viral late mRNA processing.

## Supporting information

S1 Table Amino acids with highest scores of coevolution. (TIF)

S1 Fig Schematic representation of primer design and sequences. (A) Diagram showing the L5NP and L5P primers recognition sequences (not to scale), (B) Sequences of all primers used in this work.
(TIF)

S2 Fig E1B 55K-RNP peptides do not interact with TPL RNA 20nts *in vitro*. **(**A) Expanded region of an overlay of TOCSY spectra of free WT peptide (black) and WT peptide bound to the TPL RNA 20nts probe (red). **(**B) Heat exchanged from each injection of WT peptide into a solution containing the TPL RNA 20nts probe. (C) Expanded region of an overlay of TOCSY spectra of free C287S/C288S peptide (black) and C287S/C288S peptide bound to the TPL RNA 20nts (red). (D) Heat exchanged from each injection of C287S/C288S peptide into a solution containing the TPL RNA 20nts. The thermograms were best fit to one binding site model.
(TIF)

S3 Fig Core residues stabilizing the core of the domain. Leucine in grey, isoleucine in black, phenylalanine in cyan and cystein in red, in spacefilling representation. Residues marked with a white asterisk are part of the putative RNP motif (F285, C287, and C288).
(TIF)

S4 Fig **Molecular surface representation for the wildtype (left) and RNP deletion mutant (right), with PB3 facing the viewer and the N-terminus of the domain on top.** The beta-helix turn with the deletion is rendered as a space-fill model, in CPK colors (carbon in cyan, nitrogen in blue, oxygen in red and sulfur in yellow). The putative RNP is visible (Y286 to W289). Note the equivalent position of positive charges at the lefthand side of the domain, and the occlusion of the peptide in the deletion mutant near the T2 side of the domain.
(TIF)

S5 Fig **Electrostatic potential mapped at the molecular surface for the wildtype (top row) and RNP deletion mutant (bottom row)**. The color scale spans from +5 kT/e in blue to -5 kT/e in red. The orientation of the domain is the same as in panels A and B, with the N-terminus on top and C-terminus at the bottom.
(TIF)

S6 Fig Sample of the classes of RNA-protein poses found for the RNP deletion mutant on the PB3 face, showing different angles of interaction between dsRNA and the long axis of the domain. The top row shows poses that use the remaining RBD as a binding surface; the bottom row shows a representative structure of poses that use the N-terminal helix instead. The protein is depicted as a translucent cyan ribbon, with the mutant RNP in a spacefilling representation and CPK colors. dsRNA is shown in sticks with CPK colors (C in cyan, N in blue, O in red, S or P in yellow).
(TIF)

S7 Fig Sample of the classes of RNA-protein poses found for the E1B RNP deletion mutant on the PB1 face, showing different angles of interaction between dsRNA and the long axis of the domain. The protein is depicted as a translucent cyan ribbon, with the mutant RNP in a spacefilling representation and CPK colors. dsRNA is shown in sticks with CPK colors (C in cyan, N in blue, O in red, S or P in yellow).
(TIF)

S8 Fig Sample of the classes of RNA-protein poses found for the E1B RNP deletion mutant on the T3 loop, showing two different angles of interaction between dsRNA and the long axis of the domain. The protein is depicted as a translucent cyan ribbon, with the mutant RNP in a spacefilling representation and CPK colors. dsRNA is shown in sticks with CPK colors (C in cyan, N in blue, O in red, S or P in yellow).
(TIF)

S9 Fig Deletion of the E1B 55K RNP motif reduces repression of IFIT2 expression. HFF cells treated with 1 U/ml of IFNB1 were infected with the indicated viruses or mock-infected and harvested at 8 or 36 hpi. Total RNA was isolated to prepare cDNA specific for IFIT2 and β actin, and quantified by qPCR. β actin was used as endogenous control and the samples were quantified relative to the mock-infected samples. Mean values and standard deviations from the relative quantification were plotted from two independent experiments performed in triplicate. **** P = 0.0007.
(TIF)
