# Supplementary figures and images for: The human adenovirus type 5 E1B 55kDa protein interacts with RNA promoting timely DNA replication and viral late mRNA metabolism
Source: PLoS One. 2019 Apr 3;14(4):e0214882. doi: 10.1371/journal.pone.0214882 (PMC6447194; doi:10.1371/journal.pone.0214882)

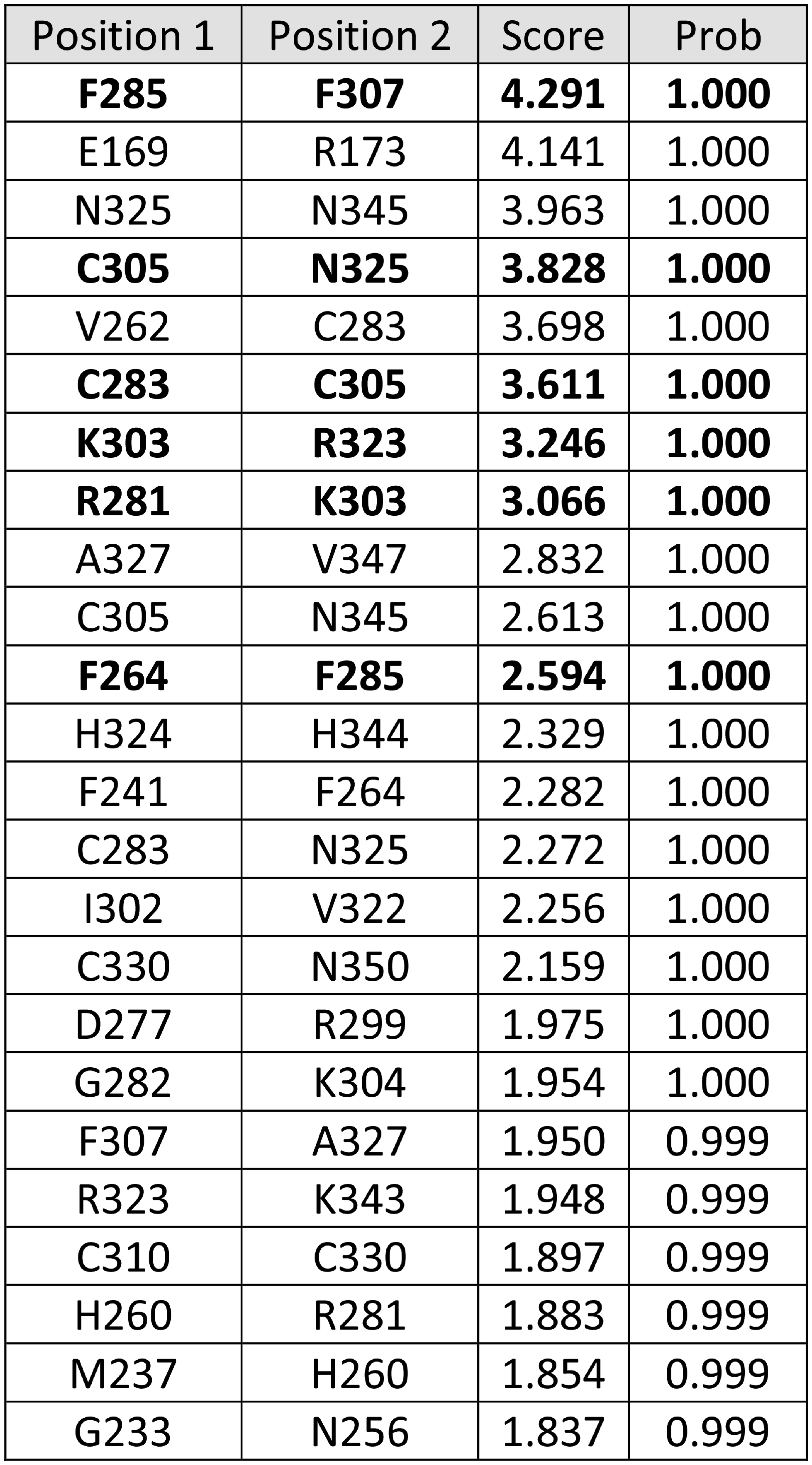

Supplement: S1 Table — (TIF) [file pone.0214882.s001.tif]

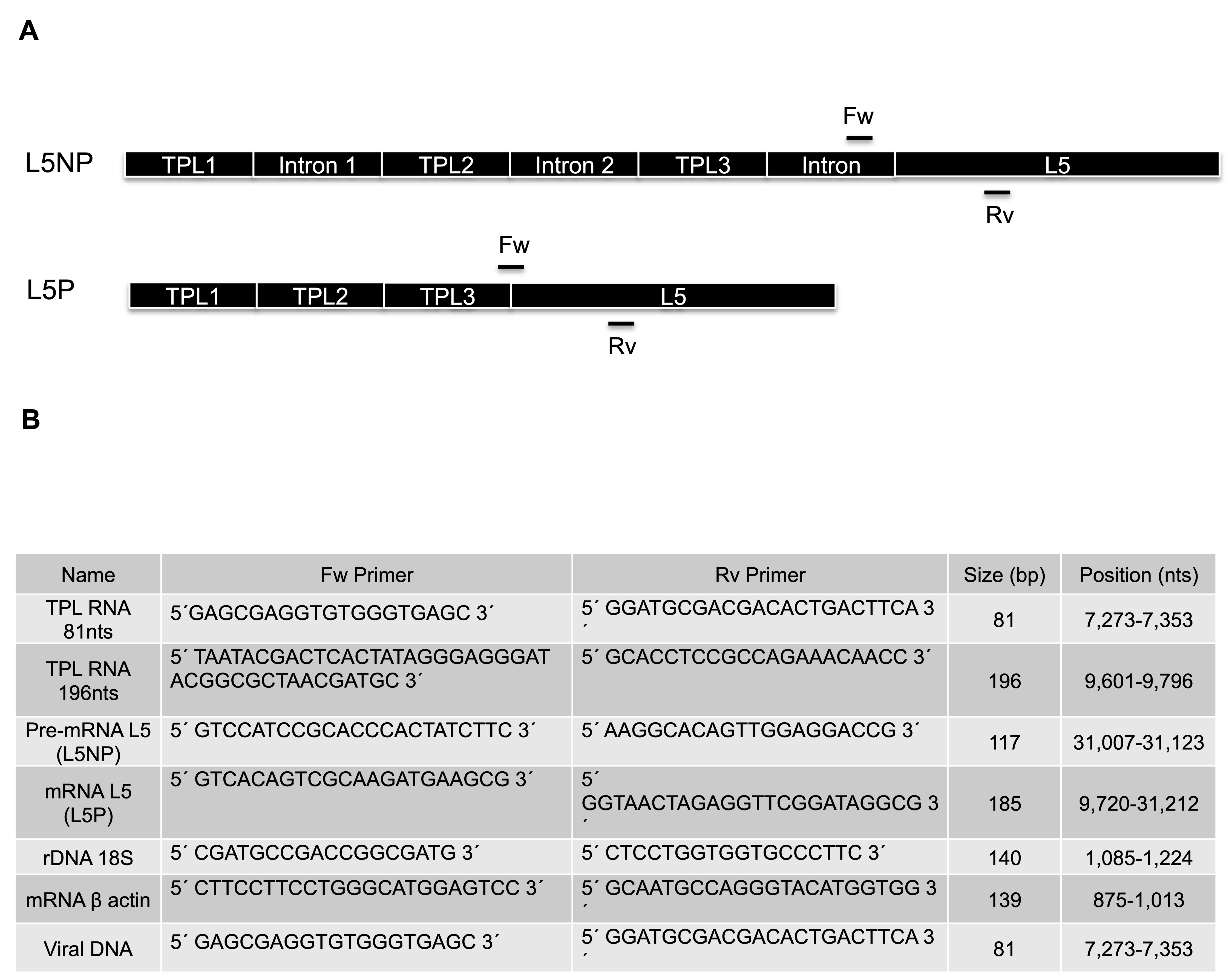

Supplement: S1 Fig — (A) Diagram showing the L5NP and L5P primers recognition sequences (not to scale), (B) Sequences of all primers used in this work. (TIF) [file pone.0214882.s002.tif]

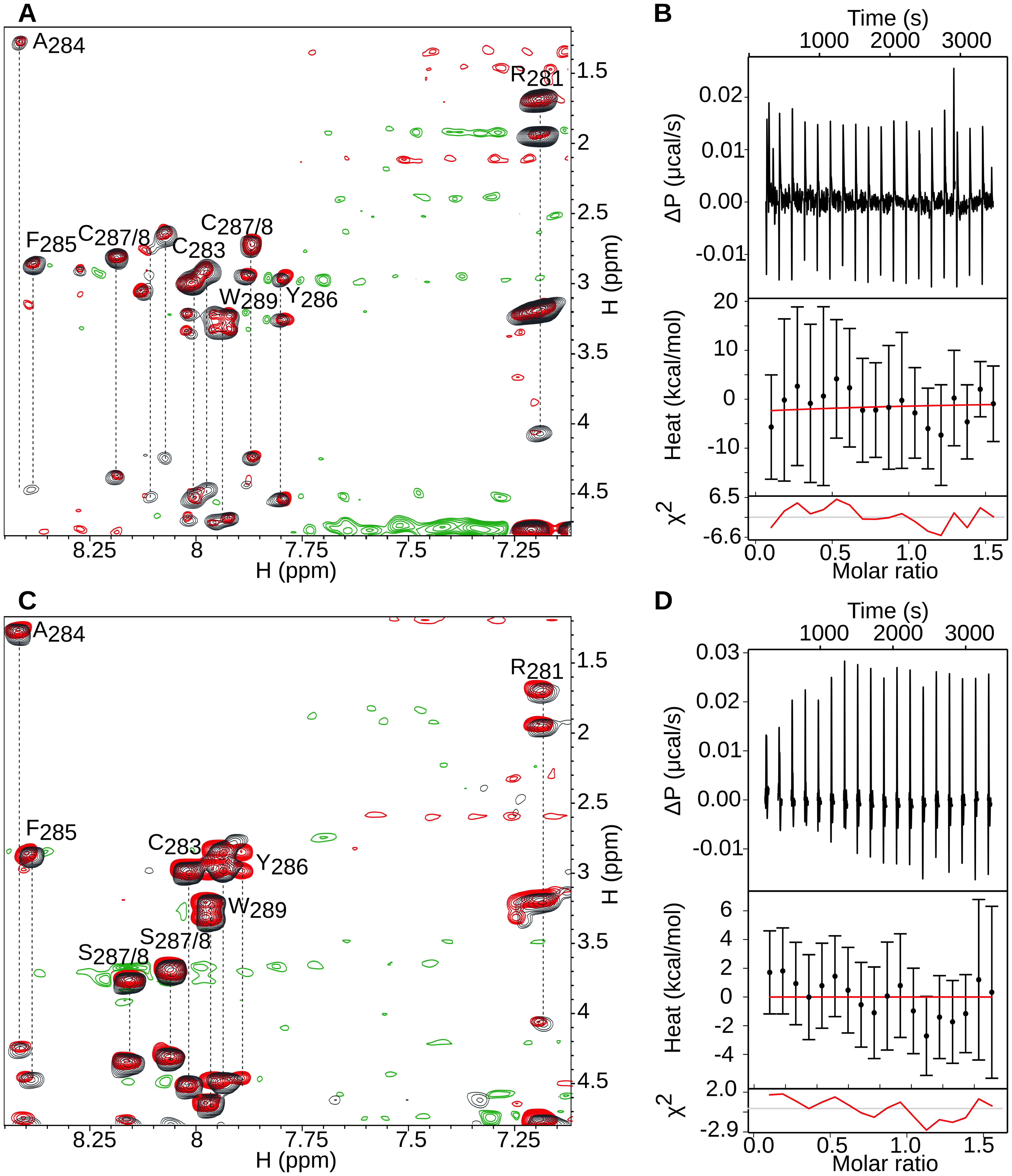

Supplement: S2 Fig — (A) Expanded region of an overlay of TOCSY spectra of free WT peptide (black) and WT peptide bound to the TPL RNA 20nts probe (red). (B) Heat exchanged from each injection of WT peptide into a solution containing the TPL RNA 20nts probe. (C) Expanded region of an overlay of TOCSY spectra of free C287S/C288S peptide (black) and C287S/C288S peptide bound to the TPL RNA 20nts (red). (D) Heat exchanged from each injection of C287S/C288S peptide into a solution containing the TPL RNA 20nts. The thermograms were best fit to one binding site model. (TIF) [file pone.0214882.s003.tif]

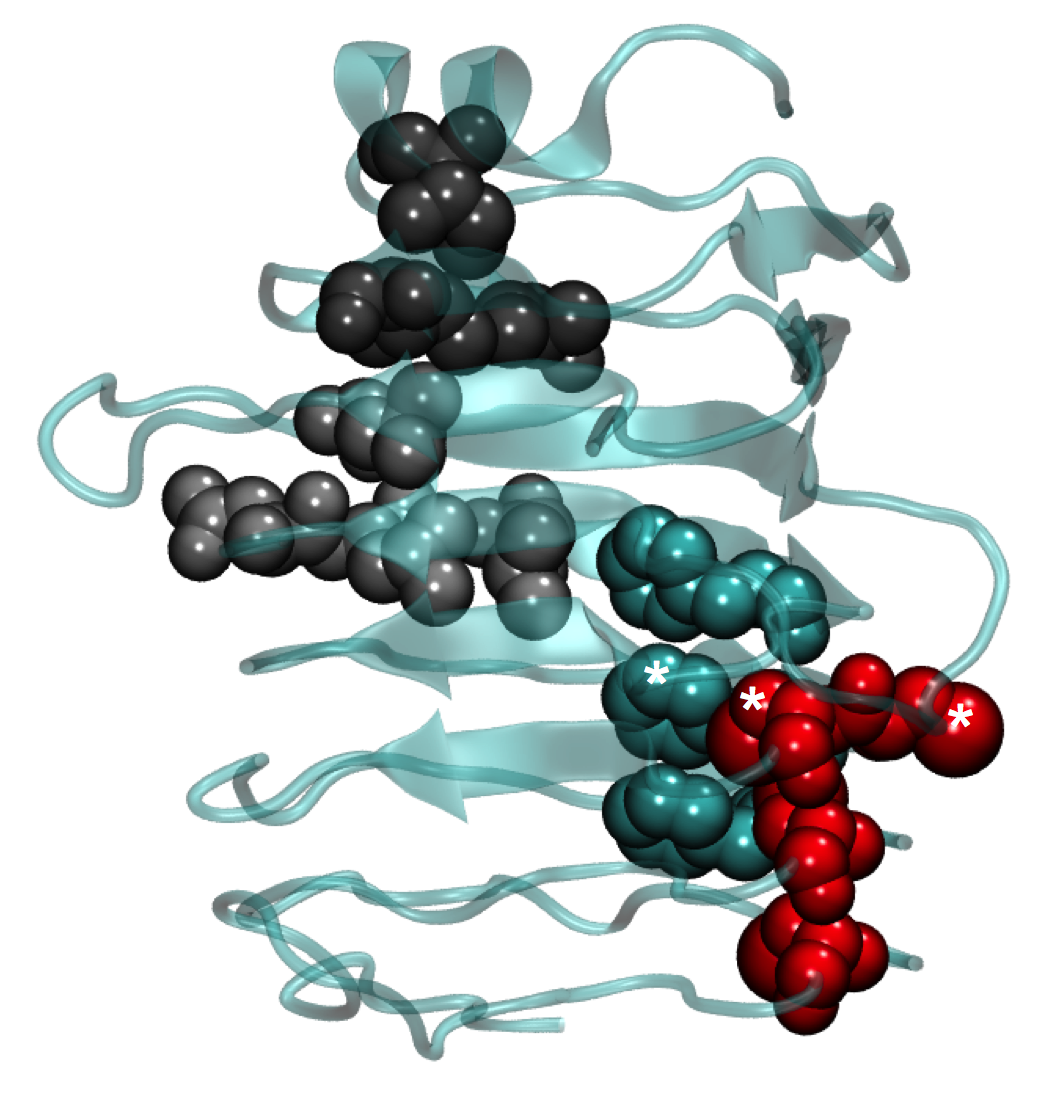

Supplement: S3 Fig — Leucine in grey, isoleucine in black, phenylalanine in cyan and cystein in red, in spacefilling representation. Residues marked with a white asterisk are part of the putative RNP motif (F285, C287, and C288). (TIF) [file pone.0214882.s004.tif]

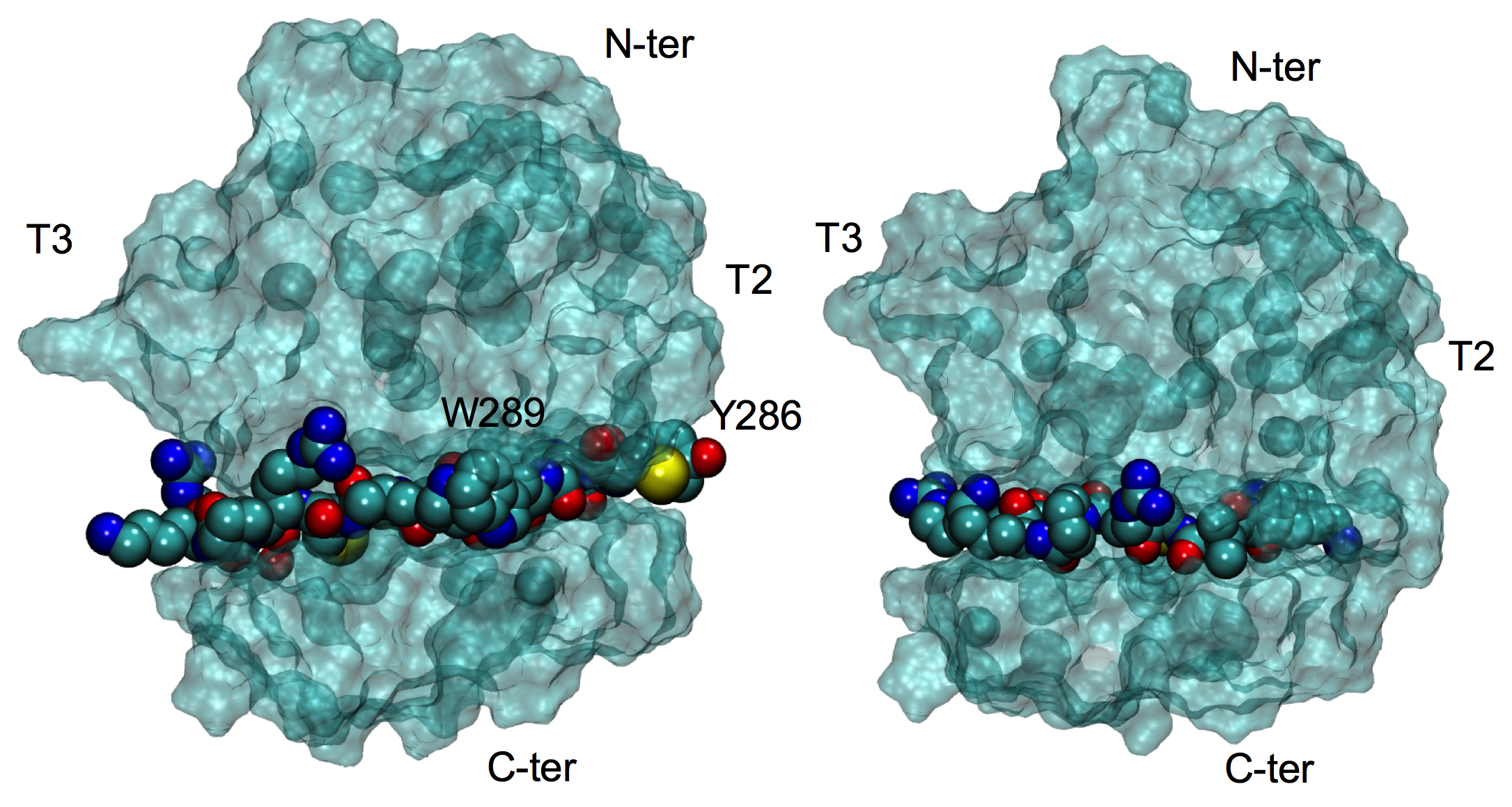

Supplement: S4 Fig — Molecular surface representation for the wildtype (left) and RNP deletion mutant (right), with PB3 facing the viewer and the N-terminus of the domain on top. The beta-helix turn with the deletion is rendered as a space-fill model, in CPK colors (carbon in cyan, nitrogen in blue, oxygen in red and sulfur in yellow). The putative RNP is visible (Y286 to W289). Note the equivalent position of positive charges at the lefthand side of the domain, and the occlusion of the peptide in the deletion mutant near the T2 side of the domain. (TIF) [file pone.0214882.s005.tif]

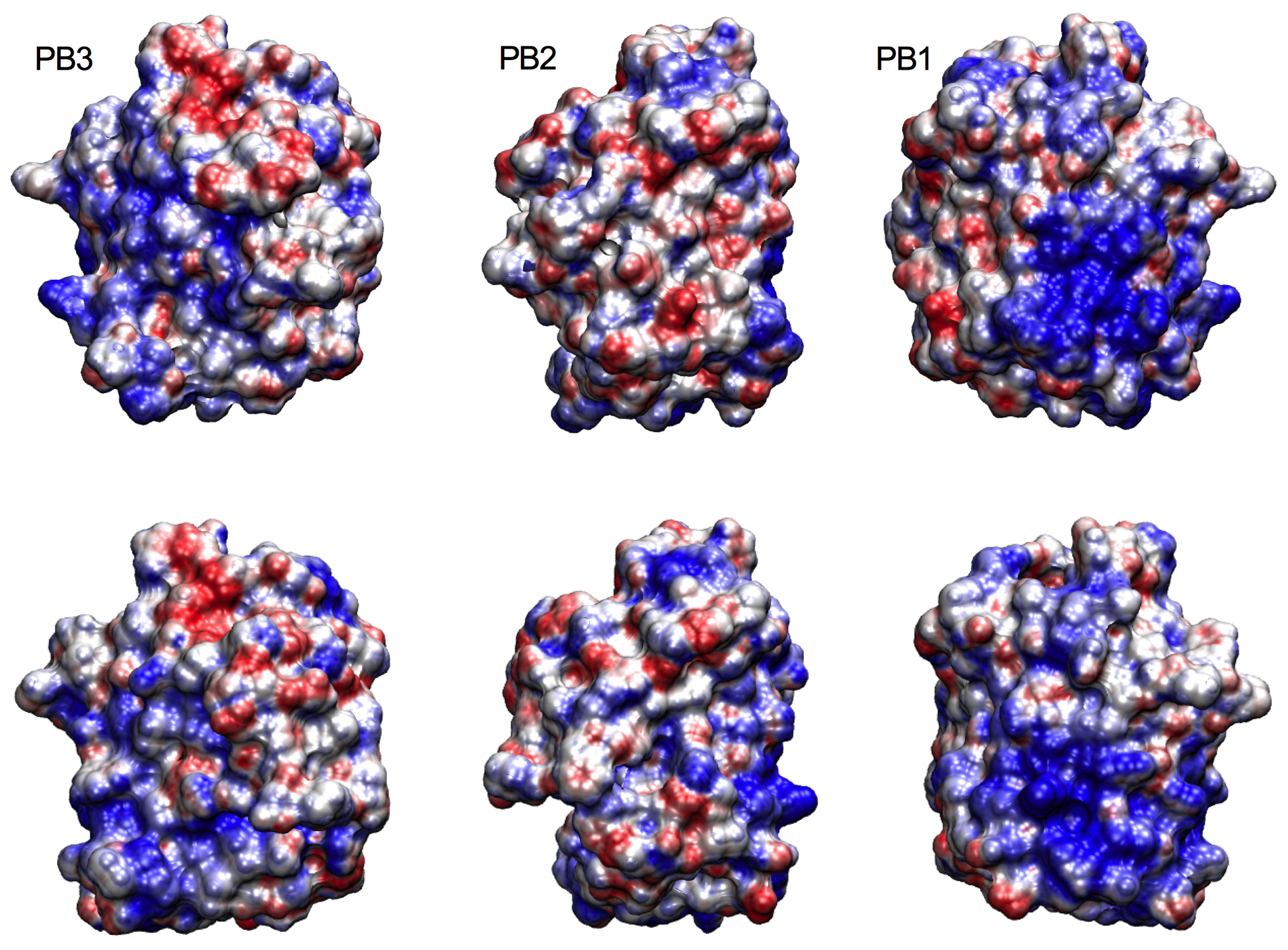

Supplement: S5 Fig — Electrostatic potential mapped at the molecular surface for the wildtype (top row) and RNP deletion mutant (bottom row). The color scale spans from +5 kT/e in blue to -5 kT/e in red. The orientation of the domain is the same as in panels A and B, with the N-terminus on top and C-terminus at the bottom. (TIF) [file pone.0214882.s006.tif]

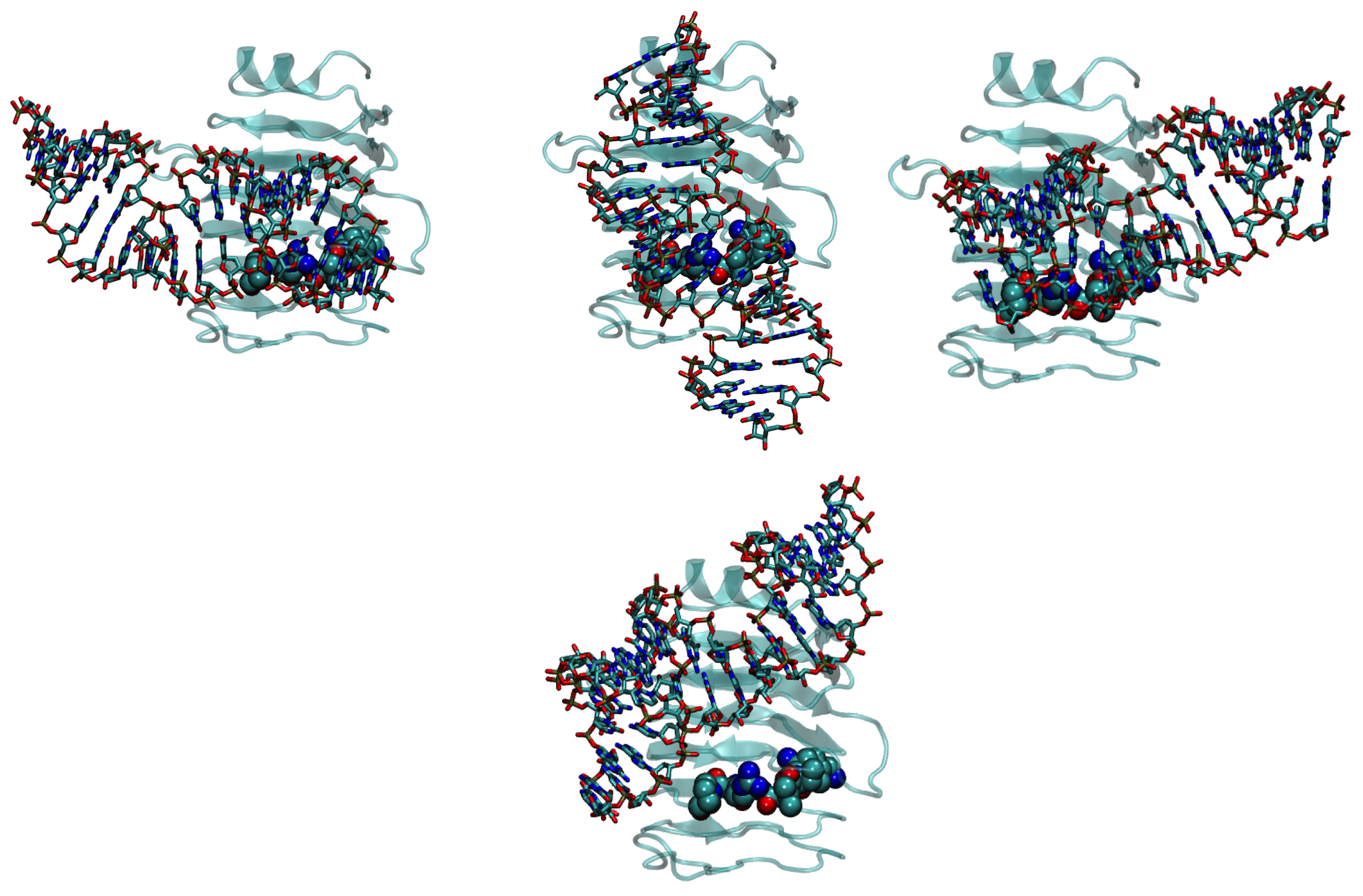

Supplement: S6 Fig — The top row shows poses that use the remaining RBD as a binding surface; the bottom row shows a representative structure of poses that use the N-terminal helix instead. The protein is depicted as a translucent cyan ribbon, with the mutant RNP in a spacefilling representation and CPK colors. dsRNA is shown in sticks with CPK colors (C in cyan, N in blue, O in red, S or P in yellow). (TIF) [file pone.0214882.s007.tif]

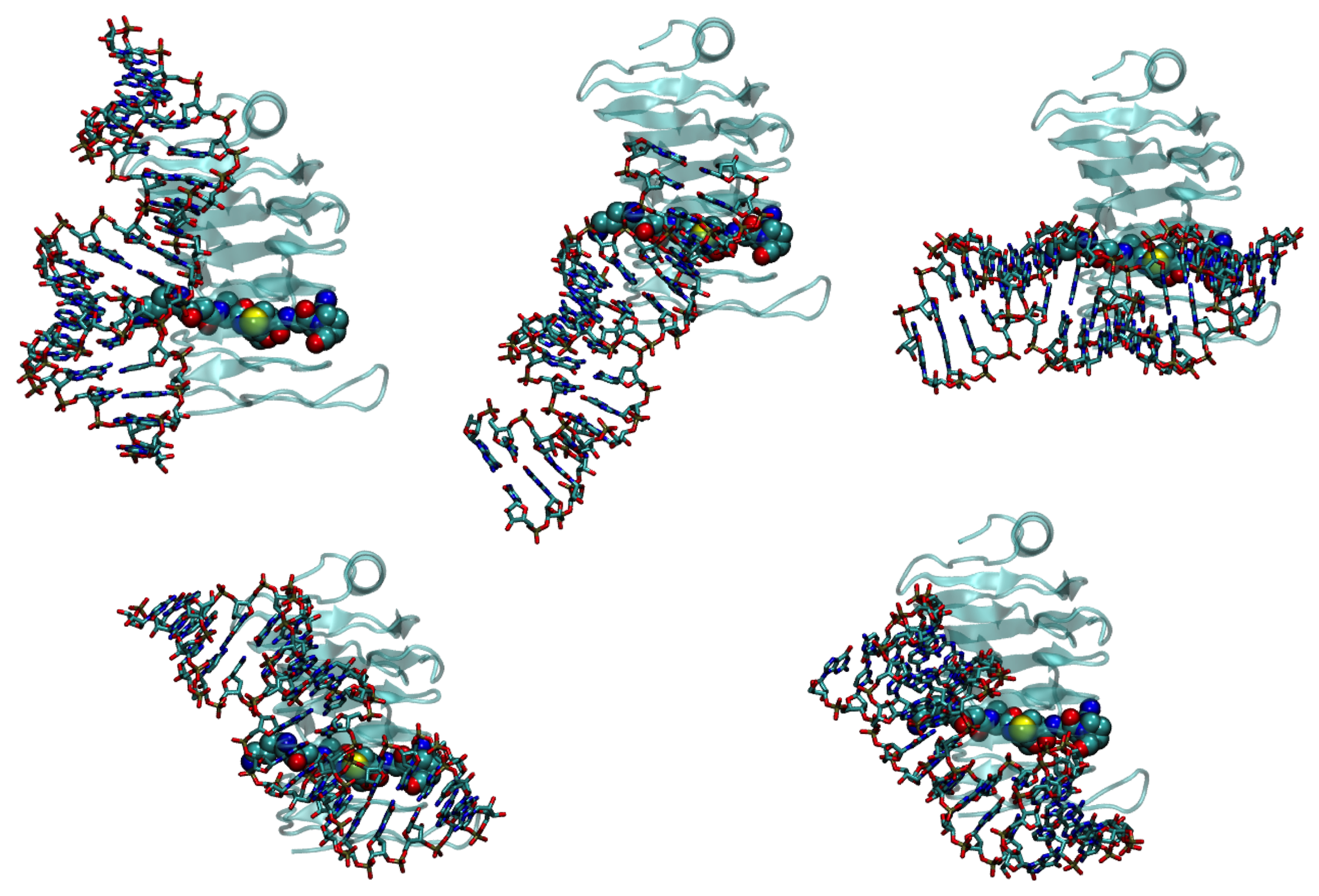

Supplement: S7 Fig — The protein is depicted as a translucent cyan ribbon, with the mutant RNP in a spacefilling representation and CPK colors. dsRNA is shown in sticks with CPK colors (C in cyan, N in blue, O in red, S or P in yellow). (TIF) [file pone.0214882.s008.tif]

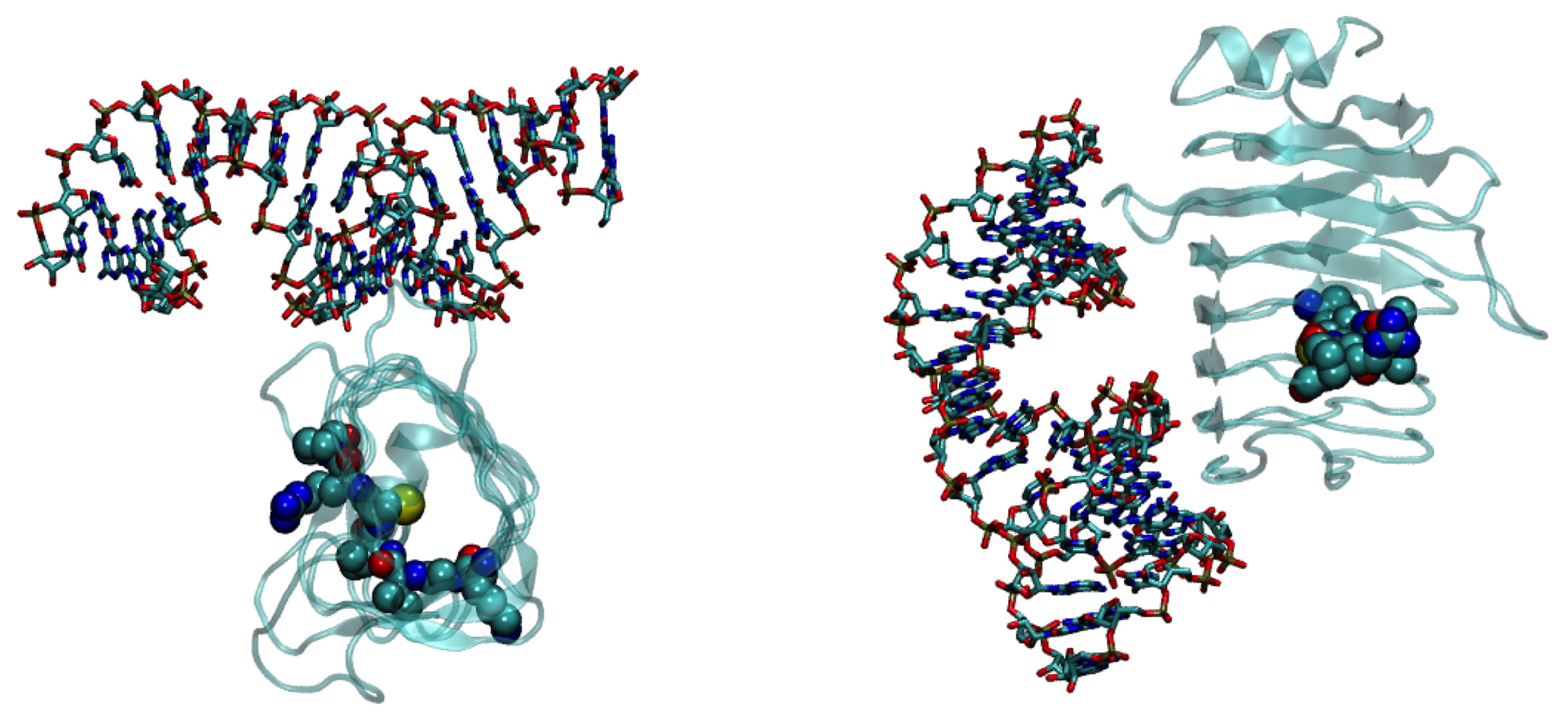

Supplement: S8 Fig — The protein is depicted as a translucent cyan ribbon, with the mutant RNP in a spacefilling representation and CPK colors. dsRNA is shown in sticks with CPK colors (C in cyan, N in blue, O in red, S or P in yellow). (TIF) [file pone.0214882.s009.tif]

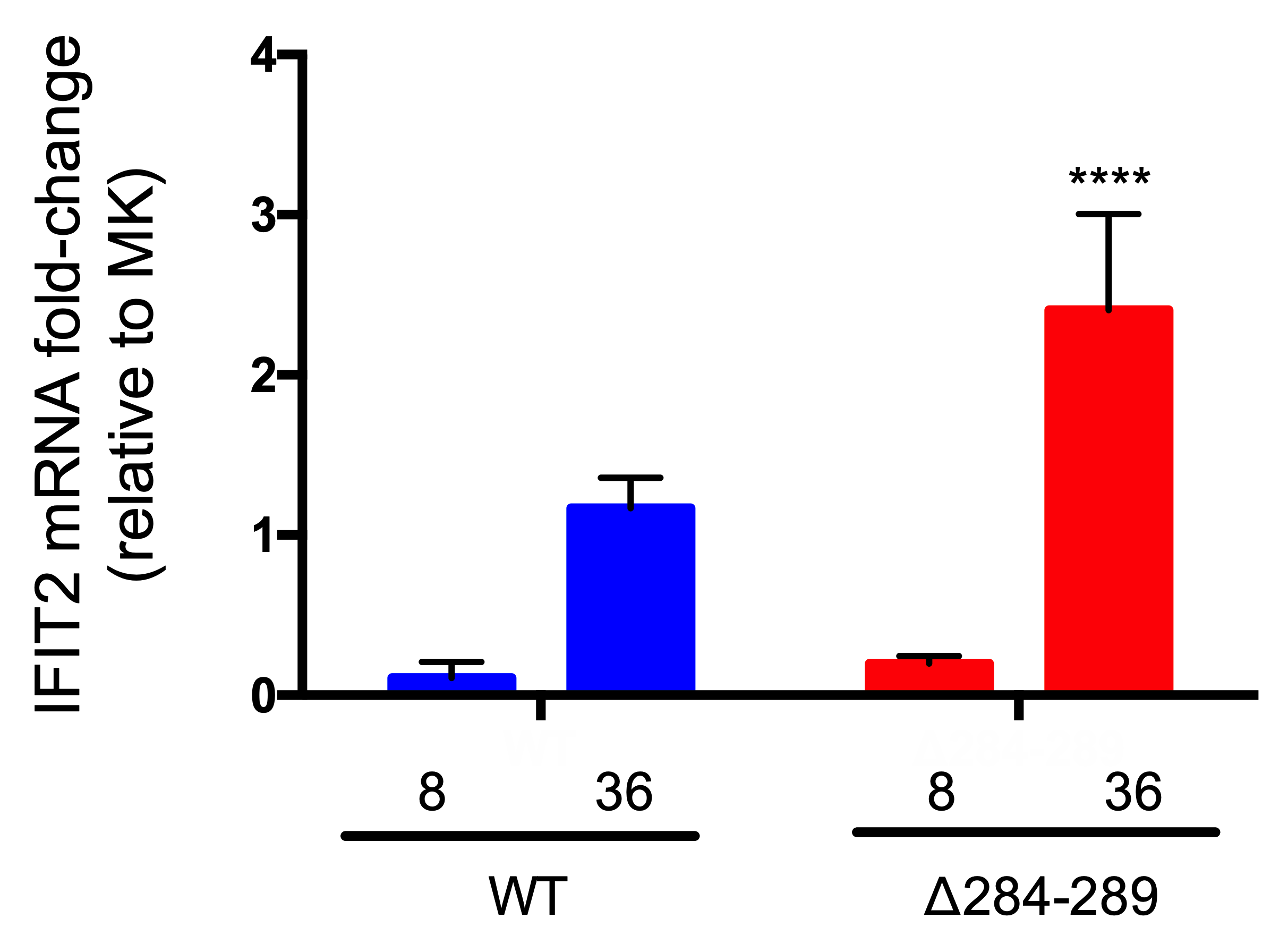

Supplement: S9 Fig — HFF cells treated with 1 U/ml of IFNB1 were infected with the indicated viruses or mock-infected and harvested at 8 or 36 hpi. Total RNA was isolated to prepare cDNA specific for IFIT2 and β actin, and quantified by qPCR. β actin was used as endogenous control and the samples were quantified relative to the mock-infected samples. Mean values and standard deviations from the relative quantification were plotted from two independent experiments performed in triplicate. **** P = 0.0007. (TIF) [file pone.0214882.s010.tif]
